# Benefits of Argan Oil on Human Health—May 4–6 2017, Errachidia, Morocco

**DOI:** 10.3390/ijms18071383

**Published:** 2017-06-28

**Authors:** Gérard Lizard, Younes Filali-Zegzouti, Adil El Midaoui

**Affiliations:** 1 University Bourgogne Franche-Comté, Lab. Bio-PeroxIL (EA7270) ‘Biochemistry of the Peroxisome, inflammation and lipid metabolism’, Inserm, Faculté des Sciences Gabriel 6, Bd Gabriel, 21000 Dijon, France; gerard.lizard@u-bourgogne.fr; 2 University Moulay Ismaïl, Research Team: ‘Biology, Environment & Health’ Department Biology, FSTE, Boutalamine, BP 509, Errachidia, Morocco; y.filalizegzouti@fste.umi.ac.ma; 3 Department Biology, FSTE–Univ. Moulay Ismaïl, BP 509 Errachidia, Morocco; adil.el.midaoui@umontreal.ca; 4 Department Molecular and Integrative Physiology, Fac. Medicine, Univ. Montréal, Montréal, QC H3T 1J4 Canada

## 1. Aim and Scope of the Meeting

The First International Symposium “***The beneficial effects of argan oil on human health***” is part of the dynamics of research and development programs on argan oil, which prompts Morocco to deploy various strategies, programs and plans for the protection and preservation of the argan tree and of its know-how that is linked to the production of argan oil. The economy associated with Argan oil concerns different sectors: agriculture, agro-industry, health and wellbeing.

The first international symposium was organized on 4, 5 and 6 May in Errachidia (Tafilalet, Morocco) on the theme “The beneficial effects of argan oil on human health” by the Research Team ‘Biology, Environment and Health’ (Univ. Moulay Ismaïl, FSTE) in partnership with the Moroccan Society of Biochemistry and Molecular Biology. Its objective was to allow researchers and experts, farmers/producers, technical institutes, decision-makers and elected representatives, to exchange about the results of research on argan oil, in the valorisation of its substances and on their chemical and biological properties.

A large call for contributions helped to harvest about a hundred contributions presented in the form of lectures (10), oral communications (22) or posters (66). The various contributions, resulting from research and R & D projects, from actions of institutions, laboratories, non-governmental organizations, international and national projects, were entirely or partially dedicated to the importance of argan oil as food with benefits to human health. People from Morocco, and North Africa (Tunisia, Algeria), Europa (France, Spain) and USA attended the meeting.

The holding of this congress in the city of Errachidia, capital of the Tafilalet region, was an opportunity for exchanges and contacts between Moroccan researchers and the international scientific community. The presence of renowned speakers nationally and internationally recognized honored and enriched this event. 

The purpose of this meeting was to provide a valuable opportunity for participants to exhibit and discuss their research and introduce new technologies. The themes addressed current international issues and raise awareness of the uses and perspectives of research on the beneficial effects of argan oil.

Below, you will find the abstracts grouped as invited presentations, oral presentations and poster presentations

## 2. Invited Presentations

### Argan Oil-Enriched Nanomedicines to Enhance Both the Efficacy and Safety of Cancer Therapy

BAYOUMIPr Tamer ELCo-Directors of the Nanomedicine Center of Excellence in Translational Cancer Research, College of Pharmacy-Glendale, Midwestern University, Arizona, USA

The unique quality of poly-unsaturated fatty acids contained in argan oil (Argania spinosa), in addition to the abundance of active compounds (e.g., sterols, carotenoids, xanthophyls, and potent antioxidants) in the un-saponifiable fraction indicate marked potential nutritional benefits for the prevention of cardiovascular diseases and cancer. As poly-unsaturated fatty acids (PUFA) have demonstrated synergism with both radio- and chemo-therapy, in solid and haematological tumors, tocopherols and saponins derived from A. spinosa exerted evident anti-proliferative effect, both in vitro and in vivo. Within past few years, the succinate ester form of α-tocopherol, d-α-tocopheryl polyethylene glycol 1000 succinate (TPGS), a common surfactant in various nanocarriers, has received notable attention for its pro-apoptotic activity against cancer, not normal, cells.

Our recent preclinical cancer data indicate that active argan oil composition displayed cytotoxic activity, when optimally formulated in a pharmaceutical nanoemulsion (NE) platform. Enhanced inhibitory effect on the proliferation of model cancer cell lines was achieved via the incorporation of active TPGS in the emulsification of argan oil NE, suggesting a cooperative role of tocopherols along with other core active oil compounds, contributing to augmented inhibition of cancer growth.

Additionally, utilizing antioxidant argan oil NE formulations—further enriched with oxygen—radical scavengers/anti-inflammatory molecules—demonstrated superior in vitro cardio-protective activities, as well as marked reduction of H_2_O_2_—and adriamycin-induced apoptotic effects. Hence, our enriched argan NEs effectively diminished oxidative and non-oxidative damage of cardiomyocytes and aortic medial cell cultures. 

Collectively, optimized nano-formulations of the unique components of argan oil (PUFAs, sterols, polyphenols, tocopherols and saponins) offers not only superior pharmaceutical properties but can also enhance its pharmacological activity profiles in both cancer and cardio-protective therapies.

### Beneficial Effects of Argan Oil on Cardiovascular Risk Factors

MIDAOUIPr Adil ELAssociate Member of the Research Team in “Biology, Environment & Health” Department of Biology; FST-Errachidia, Morocco, Associate Researcher; Department of Molecular and Integrative Physiology, Faculty of Medicine, University of Montreal, Canada

**Objective:** The present study was designed to examine the effects of argan oil on two cardiovascular risk factors notably arterial hypertension and insulin resistance as well as on the aortic basal superoxide anion production and NADPH oxidase activity in one nutritional model of hypertensive and insulinoresistant rat. 

**Methods:** Sprague-Dawley rats had free access to a drinking solution containing 10% D-glucose or tap water (control) for 5 weeks. The impact of argan oil was compared to that of corn oil given daily by gavage (5 mL/kg) during 5 weeks in glucose-fed rats. Oxidative stress was evaluated by measuring the superoxide anion production and the NADPH oxidase activity using the lucigenin method. 

**Results:** Five weeks treatment with glucose led to increases in systolic blood pressure, plasma glucose and insulin levels and insulin resistance index in association with a rise in superoxide anion production and NADPH oxidase activity (sensitive to diphenyleneiodonium) in the aorta. The simultaneous treatment with argan oil prevented or significantly reduced all those effects, yet the same treatment with corn oil had a positive impact only on hyperinsulinemia and insulin resistance. 

**Conclusions:** These findings demonstrate that argan oil treatment reduced the elevation in blood pressure, hyperglycemia and insulin resistance through its anti-oxidative properties in glucose-fed rats. Hence argan oil which is now available in the market as consumable food may be of potential therapeutic value in the treatment of arterial hypertension and insulin resistance. 

### Efficacy of Argan Oil on Pain, Metabolic Syndrome and Oxidative Stress in Rheumatology and Nephrology Patients

ERRASFAPr MouradProfesseur de l’Enseignement Supérieur de pharmacologie, Faculté de Médecine et de Pharmacie Fès. Directeur du laboratoire: Bases Moléculaires en Pathologie Humaine et Outils Thérapeutiques

Argan Oil is produced from kernel fruit of argan tree that is endemic in Morocco. The oil is used in traditional cuisine and also in traditional medicine to treat some skin and joint pain issues. 99% of argan oil composition is made of triglycerides (45% of oleic acid and 34% of linoleic acid), whereas the remaining 1% contains very active biological compounds such as Vitamine E, Carotenoïds, Sterols and Polyphenols. The above composition of argan oil is behind the many health benefits that are described in the literature, mainly on the cardiovascular system, pain-associated osteoarthritis and other metabolic parameters. In the present work, we report data on the improvement of clinical and metabolic parameters in patients upon argan oil consumption. 

**Introduction:** (1) Argan oil consumption is known to have the following properties: Lipid lowering effect in dyslipidemic patients; (2) Prevents prothrombotic complications in patients; Antidiabetic and antihypertensive effect in animal models; (3) Modulate insulin resistance and glucose intolerance in animal models 

**Aim of the study:** To investigate the effects of argan oil consumption on pain and metabolic syndrome in knee osteoarthritis patients, and to check its effects on oxidative stress and lipid profile in hemodialysis patients.

**Results:** As reported in the published articles below in reference’s section, consumption of argan oil during 5 to 8 weeks, resulted in:

- an improvement of metabolic syndrome parameters, pain score and walking difficulties in rheumatology patients.
- Improvement of blood lipids and atherogenic lipid ratios, as well as oxidative stress parameters in hemodialysis patients.

**Conclusions:** (1) Argan Oil can be used as a therapeutic «green medicine» in various health issues. (2) Promotion of clinical studies on Argan Oil should help understanding more of its health benefits in other pathologies. (3) Medical investigations on Argan Oil health benefits should boost all Argan oil-related activities such as women cooperatives associations, organic farmers, and Argan Oil quality assurance.

### Efficacy of a New Pharmaceutical Formulation of Melatonin in Preventing Aging Skin Damage

ESCAMESPr GermaineResearcher in Biomedical Research Centre, Department of Physiology, Faculty of Medicine, Granada, Spain

Melatonin, a hormone produced by the pineal gland, has been detected in multiple extrapineal organ tissues at much higher concentrations than in the pineal gland. It is a potent free radical scavenger with anti-oxidant properties, which increases the expression and activity of endogenous antioxidant. This special class of antioxidant generates a series of metabolites that are also free radical scavengers when scavenging free radicals. Capable of crossing cell membranes and of easily reaching all cell compartments, it is taken up by mitochondria and can maintain mitochondrial homeostasis in different experimental models. Melatonin has also important anti-inflammatory effects.

Recently, we have developed a pharmaceutical preparation of melatonin plus other molecules for the treatment and prevention of skin aging. The success of this preparation is that the composition facilitates their transdermal adsorption, reaching both molecules all skin’s layers. Moreover, the combination of both molecules increases their take up by the mitochondria in all skin cells. The advantage of our product is that not only reverse the mitochondrial damage produced during cellular aging but also in many pathologies coursing with mitochondrial impairment.

### Interest of Argan Oil for The Prevention of Neurodegenerative Diseases: In Vitro and In Vivo Proofs of Concept

LIZARDDr GérardDirecteur du Laboratoire Bio-PeroxIL / Chercheur Inserm: Biochimie du Peroxysome, Inflammation et Métabolisme Lipidique, Université de Bourgogne/Inserm, Dijon, France

In major age-related diseases such as cardiovascular diseases, certain ocular diseases (cataract, age-related macular degeneration) and neurodegenerative diseases (particularly Alzheimer’s disease), a rupture of the RedOx equilibrium is observed. This enhances the lipid peroxidation associated with aging and increases the formation of certain oxidized cholesterol derivatives, called oxysterols, especially those formed by auto-oxidation such as 7-ketocholesterol (7KC). This lipid peroxidation is also accompanied by a degradation of the unsaturated fatty acids which generate highly reactive aldehydes which can lead to the formation of carbonylated proteins whose activities are modified compared to those of the native proteins from which they are derived. This results in perturbations of cell signaling leading to several cellular dysfunctions. Furthermore, the ability of molecules derived from lipid peroxidation to stimulate oxidative stress and/or inflammation, alter cell metabolism and induce changes in DNA may result in cell death. 7KC is the oxysterol preferentially formed by auto-oxidation of cholesterol. Since 7KC is weakly metabolized (at the exception of its degradation in bile acids by liver cells), it accumulates progressively, mainly in brain cells, and may induce cytotoxic effects: oxidative stress, inflammation and cell death. Its contribution to age-related diseases is therefore widely suspected. In order to prevent the cytotoxic effects of 7KC on the nerve cells of the central nervous system, one of the possibilities is to identify cytoprotective agents in order to reduce or inhibit its harmful activities. The cytoprotective agents may be either natural or synthetic molecules or mixtures of molecules such as oils. In this context, the cytoprotective properties of argan oils (Agadir, Berkane) and some of their major compounds (especially α-tocopherol) have been studied. To this end, complementary techniques of microscopy, flow cytometry and biochemistry were used. The composition of Argan oils has also been determined by different chromatography techniques. Argan oils have highly antioxidant properties even in vivo when given per os in the rat. On 158N murine oligodendrocytes, these oils are capable of attenuating the cytotoxicity of 7KC: loss of cellular adhesion, alteration of the plasma membrane, mitochondrial and lysosomal dysfunctions, overproduction of oxygen radicals, induction of apoptosis and autophagy. Overall, our findings provide several in vitro and in vivo proofs of concept that suggest that Argan oils could prevent and mitigate severe age related pathologies especially neurodegenerative diseases.

## The following abstracts were submitted in French and only displayed by Title, Authors and Affiliations.

### Mediterranean Diet and Coronary Disease: Potential Beneficial Effects of Argan Oil

LEIRISPr Joël deChercheur au laboratoire Cœur et Nutrition, Unité Mixte de Recherche Techniques de l’Ingénierie Médicale et de la Complexité—Informatique, Mathématiques et Applications, Grenoble, France

### The Neuro-Cardiovascular and Androgenic Benefits of Argan Oil in Humans

DEROUICHEPr AbdelfettahResponsable d’unité Nutrition Humaine, Laboratoire de Recherche sur les Lipoprotéines et l'Athérosclérose, Faculté des Sciences Ben M'Sik, Université Hassan II Mohammedia, Casablanca. Coordinateur national pour l'Action Mondiale pour le Sel et la Santé (Word Action on Salt & Health: WASH), Vice-Président: de la Société Marocaine de Nutrition: La Nutrition saine pour tous et de l’Association Tous Avec le Coeur: La prévention primaire des Maladies Cardiovasculaires

### The Aqueous Extract of Argania spinosa Attenuates Streptozotocin-Induced Diabetes Mellitus in Rats

EDDOUKSPr MohamedResponsable de l’équipe de Recherche Physiologie & Pharmacologie Endocrinienne. Il a exercé les missions de doyen de la Faculté polydisciplinaire d’Errachidia (2008–2012) et Vice-doyen chargé de la recherche scientifique à la FST Errachidia (2005–2008). Il a été classé 2ème à l'échelle du monde arabe dans le domaine des sciences pharmaceutiques. Il a été choisi également parmi les trois meilleurs au TOP 5 des chercheurs marocains les plus actifs dans le domaine des sciences de la vie «Life Sciences» par l’Institut marocain de l'information scientifique et technique (IMIST)

### Chemical Characterization and Description of New Aminophenolic Compounds of the Fruit of the Moroccan Argan Tree

KHALLOUKIPr FaridChercheur au Département de Biologie, Faculté des Sciences Techniques, Errachidia, Maroc

### Neuroprotective Activities of Argan Oil against Pathologies of the Nervous System

MESFIOUIPr AbdelhalimResponsable de l’équipe Biotechnologie et Valorisation des Bioressources, Laboratoire de Génétique —NeuroEndocrinologie—Biotechnologie, Faculté des sciences Kenitra, Maroc

## 3. Oral Presentations Theme: Chemical Structures, Properties and Additional Value of Argan.

### Comparison of the Contents of the Main Biochemical Compounds and the Antioxidant Activity of Argan Oil, Olive Oil, Silybum Marianum Seed Oil, Nigella Seed Oil and Colza Oil

ZarroukA.^1^,^2^,^3^MartineL.^4^GrégoireS.^4^CamusE.^5^MeddebW.^6^KarymE.-M.^7^BadreddineA.^7^HarzallahA.^1^Hadj AhmedS.^1^DurandP.^5^ProstM.^5^MejriM.^6^Cherkaoui-MalkiM.^2^NasserB.^7^HammamiM.^1^BretillonL.^4^LizardG.^2^1Laboratoire ‘Nutrition, Aliments Fonctionnels et Santé Vasculaire’, UR12ES05 Université de Monastir, Monastir, Tunisia2Equipe ‘Biochimie du Peroxysome, Inflammation et Métabolisme Lipidique’ EA 7270/Université de Bourgogne Franche-Comté/Inserm, Dijon, France3Laboratoire de Biochimie, Faculté de Médecine, Sousse, Tunisia4Eye and Nutrition Research Group, Centre des Sciences du Goût et de l’Alimentation, UMR 1324 INRA, 6265 CNRS, University of Bourgogne Franche-Comté, Dijon, France5Laboratoire LARA-SPIRAL, Couternon, France6Institut Supérieur de Biotechnologie, Béja, Tunisia7Laboratory of ‘Biochemistry of Neuroscience’, University Hassan I, Settat, Morocco

The beneficial activities of vegetable oils results from their components. Thus, we intended to compare phytosterols, fatty acids, tocopherols, and polyphenols contents of dietary and cosmetic argan oil (AO), olive oil (OO; Tunisia, Morocco and Spain), silybum marianum seed oils (SMSO; Zaghouane, Bizerte, Sousse), nigella seed oil (NSO) and colza oil (CO), using analytical methods. The antioxidant potential of the oils was evaluated with KRL test. Oils had high C18:2 contents, with highest amounts in SMSO and NSO. CO had the highest amount of C18:3. Its level was eight to ten folds higher in NSO compared to the AO and SMSO. OO and AO had the highest level of C18:1. OO had the highest level of C16:0. C22:0 and C24:0 were found with elevated amounts in SMSO. CO and Berkane AO were rich in γ-tocopherol. Agadir AO contained the highest amount of α-tocopherol, which was present in an appreciable level in SMSO Zaghouan and Tunisian OO. Phytosterols profile showed that β-sitosterol was the major sterol in all the oils in the exception of AO. Spinasterol and schotenol were present in an important amounts in AO. Schotenol was also detected with highest level in SMSO. Stigmasterol, ∆7 campesterol, β amyrine were detected especially, in SMSO. Polyphenol profile showed the presence of homovanillic acid, vanillin, p-Coumaric acid, quercetine-3β-glucoside, quercetin and apigenin in SMSO. In AO, only protocatechic acid and tyrosol were identified. In NSO, 2.6-dihydroxybenzoïc acid, thymoquinone homovanillic acid, vanillin were detected. The antioxidant ability of the oils showed that CO, Sousse SMSO and dietary Agadir AO had the highest Red blood cells half-hemolysis time in min, which reflect their ability to increase resistance of the cells to hemolysis. The antioxidant ability of dietary and cosmetic Berkane AO was positively correlated to procatechic acid and compestanol levels. The above-mentioned makes AO and SMSO preferable choice for diseases preventing diets.

**Keywords:** Phytosterols; fatty acids; tocopherols; polyphenols; antioxidant activity

### The Antioxidant Properties of Phenolic Compounds Found in Argan Oil. DFT/QSAR Results and Molecular Docking

ZakiH.^1^,^2^*Filali ZegzoutiY.^1^BenlyassM.^1^BouachrineM.^2^*1Research Team Biology, Environment & Health, FSTE, Moulay Ismail University, Meknes, Morocco2Research Team Materials, Environment & Modeling. ESTM, Moulay Ismail University, Meknes, Morocco*Correspondence:

The argan oil has been known for its various pharmacological properties and used as a natural remedy since several centuries. Argan oil is rich in oleic acid and linoleic acid. Interestingly, the unsaponifiable fraction of this oil is mainly rich in antioxidant compounds such as sterols, saponin and phenolic compounds, principally α-tocopherol isoform Considering its rich composition in antioxidant compounds and unsaturated fat, argan oil can be used as a nutritional intervention in the CVD and cancer disease prevention. 

In this work we attempt to establish a quantitative structure-activity relationship for antioxidant activity by studying a series of flavonoid compounds. We accordingly propose a quantitative model, and we try to interpret the activity of the compounds and predict the antioxidant activities of the phenolic compounds present in argan oil such us alpha tochoferol, gamma tochoferol and delta tochoferole relying on the multivariate statistical analyses. Also we attempt to validate the antioxidant activity of these compounds by docking study against cyclooxygenase-2 target (4COX) to predict and compare the conformations of ligands and orientations of binding properties of compounds. 

MLR has served to select the descriptors used as the input parameters the MNLR and ANN.the topological descriptors and the electronic descriptors were computed with ACD/ChemSketch and Gaussian 03W program, respectively and the Docking Study performed With Autodock Vina Programm.

**Keywords:** Argan oil; QSAR, Docking; Phenolic compounds; antioxidant activity

## Theme: Argan and Environment

### Modeling Spatial Distribution of Argania Spinosa under a Changing Climate

MoukrimS.^1^*LahssiniS.^2^ArahouM.^1^RhaziL.^1^1Mohammed V University in Rabat, Faculty of Sciences, Laboratory of Botany, Mycology and Environment, Avenue Ibn-Battouta B.P. 1014 RP, Rabat, Morocco2National School of Forest Engineer, BP 511, Tabriket-Sale, Morocco*Correspondence: maildemoukrim@gmail.com

Argan trees (*Argania spinosa*) provide many functions and ecosystem services and are an important source of income for local people. The main source of income results from fruit production from which a highly valuable oil is extracted. Current production of oil exceeds 4000 T/year. Most of the Argan populations are located in regions characterized by water scarcity and vulnerability to desertification and that will strongly be impacted by climate change. As a result, the impact on the future Argan distribution in response to climate change should be assessed. Such understanding is valuable for prioritizing short and long-term management efforts, and is very useful for supporting decisions in order to ensure a sustainable production of Argan oil.

To examine the relation between bioclimatic variables and changes in Argan distribution, species distribution models (SDM) were used. A Maximum Entropy Modeling (MaxEnt) algorithm was chosen to link species locations with environmental characteristics in order to predict species occurrence likelihood and to assess the contribution of each environmental variable.

The output from this study is a continuous probability map showing the current and predicted areas suitable for Argan trees in Morocco under different climate change scenarios. The relative contribution of each covariate to the model showed that the coldest quarter and wettest month, in addition to temperature seasonality, contribute significantly to explain the spatial distribution of Argan trees.

Using a predefined threshold, the area suitable for Argan trees distribution seems to be highly dependent on climate change with a considerable decrease in extent. Some new areas may become suitable for Argan trees in the north of the current area. However, these gains are projected to be small, since larger regions located in the southern part of the country will become less suitable.

The conclusions from this analysis may contribute to improve management strategies in order to conserve the valuable ecosystem and to improve the production of Argan oil. We suggest conservation actions in the impacted areas and tree planting in regions that may become highly suitable in the future.

**Keywords:** Argan; SDM; Morocco; Climate change; MaxEnt

### Geoclimatic Influences on Antioxidant Activity, Polyphenolic and Flavonoids Content of the Argania spinosa Pulp

ZouhairF. Z.^1^,^2^*BenaliA.^2^EnnahliY.^2^KebbourR.^2^BouksaimM.^2^EssamriA.^1^1Laboratory of Agroresources and Process Engineering, Faculty of Sciences, University Ibn Tofail, B, P14000 Kenitra, Morocco2Laboratory of Food Technology URPAF, National Institute for Agricultural Research, Rabat, Morocco*Correspondence: fatidoc.89@gmail.com

*Argania spinosa* (L.) Skeels is a tropical plant, which belongs to the Sapotaceae family. In Morocco this tree is considered an important forest species due to its botanical, social and economical interest as well as its environmental value. 

Recently, many beneficial bio-molecules compounds have been identified from various parts of the *A. spinosa*, which can play a beneficial role in fighting disease and could be used in pharmaceutical and personal care product industries.

Phenolic and flavonoids compounds, as secondary metabolites, are a large group of molecules widely distributed in plants. Phenolic compounds can play the role of antioxidants through different mechanisms; Previous research has reported that phenolic composition is vastly influenced by biotic and abiotic factors. 

The present study was conducted on the *A. spinosa* pulp to show the antioxidants levels, polyphenolic and flavonoids contents in this pulp, coming from different areas of Morocco, and compared them with each other, beside of showing the geoclimatic influences on the composition.

**Keywords:** *Argania spinosa* pulp; antioxidant; polyphenolic compound; flavonoids; Comparative study

## Theme: The Role of Argan Oil in the Prevention and Treatment of Cardiovascular Diseases

### Anti-Inflamatory Effects of Polyphenol Fractions Purified from Argan Oils

LimaE.^1^Mrani AlaouiM.^2^AlchéV.^3^Jimenez-LopezJ.C.^1^de DiosJ.^1^*1Department of Biochemistry, Cell and Molecular Biology of Plants, Estación Experimental del Zaidín (CSIC), Profesor Albareda 1, 18008 Granada, Spain2Département de Biologie, Faculté des Sciences, Université Abdelmalek Essaâdi, Tétouan, Morocco3Andalusian Health Service. Granada, Spain*Correspondence: juandedios.alche@eez.csic.es

Argan oils are becoming widely used within the complex international oils market, due to their unique organoleptic properties together with their health-promoting characteristics and their expansive dermocosmetic uses.

Polyphenols are present in argan oils in noticeable amounts, and pioneer determinations of their composition have been recently released into the literature. Such components have been claimed as putatively responsible for pharmacological properties of virgin argan oil, mainly through their action preventing the damaging effects of reactive oxygen species (ROS), involved in the pathology of numerous diseases.

We have tested here the effects of the addition of polyphenols extracted from argan oils from different uses (edible oil, cosmetic oil and beauty oil), to whole blood cultures where an inflammatory response was triggered by means of chemical inductors. Two types of patients were assayed: healthy patients and diabetics, as the later group has been described to develop subclinical inflammatory reactions. Three key markers were analysed in plasma samples after culture by using Western blotting approaches: presence of iNOS and IL-1β, and finally the plasma SDS-PAGE profiles relatives to protein-bound 3-nitrotyrosine, as a marker of inflammation and NO production. 

The three markers were enhanced in inflammation-induced samples in comparison with controls. Challenge with argan oil polyphenols together with the induction resulted in significantly lower enhancement for both diabetic and healthy patients. Finally, the argan oil polyphenols themselves did not have inflammatory effects. 

The described effects are promising for the definition of health-promoting effects of argan oil, and the development of new therapeutically valuable tools. However, much progress is needed in order to dissect the precise causes of the described effects. 

**Keywords:** argan; anti-inflammatory; cytokines; oil; polyphenols 

This work was supported by ERDF-cofinanced research grants BFU2016-77243-P, P2011-CVI7487, 201540E065, RTC-2015-4181-2 and RTC2016-4824-2.

## Theme: Implications of Argan Oil in the Prevention and Treatment of Different Pathologies

### Potential Application of Calcium-Binding Protein of Argane Seed in Pharmacology

KarbouS.^1^,^2^,^3^,^4^*El BahloulY.^1^BenajiB.^4^BouksaimM.^3^TaghoutiM.^3^TaoudiM.^2^1UR Amélioration des Plantes Conservation et Valorisation des Ressources Phytogénétiques -Centre Régional de la Recherche Agronomique de Rabat2Université. Hassan II-Casablanca. FSAC, Laboratoire Microbiologie, Pharmacologie, Biotechnologie et Environnement3Laboratoire Biotechnologie, Centre Régional de la Recherche Agronomique de Rabat4Ecole Normale Supérieur de l’enseignement technique Université Mohammed V Rabat*Correspondence: skarbou@gmail.com

The oleaginous seed as Argane seed constitutes the source of many interesting components having great nutritional, cosmic and medical values.

It is noticed that the Argane oil is known already plays a beneficial role to prevent human health against many diseases as cardiovascular diseases. Also, the qualified antistress molecules may be playing an important role in medical field, as Calcium-binding proteins, which we have recently highlighted in Argane seed.

Our recent biochemical analysis of Argane seed proteins, showed that a protein type Calcium-binding present the instantaneous function to solubilize calcium phosphate microgranules by surrounding them in a micellar structure as other proteins types binding calcium such as caseins protein (Holt C et al., 2013). 

At all, according to our result, a potential application of calcium binding protein from Argane seed may be used against crystals Calcium phosphate formation that can cause many diseases as severe inflammation and Osteoarthritis.

**Keywords:** Argane seed; Calcium-binding protein; Calcium phosphate; diseases 

### Protective Effect of Argan Virgin Oil on Titanium Dioxide Nanoparticles Induced Toxicity and Oxidative Stress in Wistar Rats

BakourM.*ImtaraH.SouloN.LyoussiB.Laboratory of Physiology-Pharmacology-Environmental Health, Faculty of Sciences Dhar El Mehraz, University Sidi Mohamed Ben Abdallah, Fez, Morocco*Correspondence: meryem.bakour@usmba.ac.ma

Dioxide nanoparticles (TiO2NPs) are used in widespread applications, such as, drug additives, cosmetics, paints, paper, inks, sun-screens, electronics and food. The main factor in the toxicity of nanoparticles is its size which is very small and which makes it capable of inducing damage even at the level of the DNA. Thus, the aim of the present study was to investigate the protective effect of argan virgin oil on titanium dioxide nanoparticles toxicity.

Rats were randomly divided into four groups (*n* = 5), tree groups are controls the first one received (10 mL/kg b.w) of distilled water , the second exposed to (TiO2NPs) (200 mg/kg b.w) by gavage and the third received (10 mL/kg b.w) of argan oil (10 mL/kg b.w), whereas the last group is the group which is treated by the co-administration of argan oil (10 mL/kg b.w) and (TiO2NPs) (200 mg/kg b.w) for 3 weeks. Serum protein, albumin levels and transaminases (AST, ALT), ALP activities were determined, Lipid profile, urea, Creatinine, and histological changes was investigated. The obtained results indicate that the majority of the analyzed parameters were protected against the toxicity of (TiO2NPs) in the group treated with argan oil (10 mL/kg b.w).

In conclusion, our findings show that the consumption of virgin argan oil has a protective effect against the toxicity induced by titanium dioxide nanoparticles.

**Keywords:** Argan virgin oil; TiO2NPs; Toxicity

## The following abstracts were submitted in French and only displayed by Title, Authors and Affiliations.

### Physical Properties of Wood Swelling Composite Deriving from Argan Nut Shells (CNAR)

DerouicheA.BabtyF.El FassiS.MordaneS.BettachyA.Laboratoire de Physique des Polymères et Phénomènes Critiques.Université Hassan II-Casablanca, Faculté des Sciences Ben M’Sik, Casablanca

### Application of Argan Oil as a Biodegradable Inhibitor against Corrosion of Carbon Steel in an Acid Medium and Improvement of Its Anti-Corrosive Power by Enrichment in Essential Oil

TaoufikF.^1^*AnejjarA.^2^HamdouchA.^3^AsdadiA.^3^EL HadekM.^1^SalghiR.^2^ChebliB.^2^IdrissiL. M.^3^1Laboratoire de Génie des Procédés, Faculté des Sciences d’Agadir, Université Ibn Zohr, B.P 28/S, Agadir, Maroc.2Equipe de Génie de l’Environnement et de Biotechnologie, ENSA, Université Ibn Zohr, BP 1136, Agadir, Maroc3Laboratoire de Biotechnologies Végétales, Equipe Planta Sud, Faculté des Sciences d’Agadir, Université Ibn Zohr, B.P 28/S, Agadir, Maroc*Correspondence: taoufik.fatima@gmail.com 

### Effects of Drought on the Productivity of Argan Trees in the Rural Municipality of Imin'tlit (Province of Essaouira)

AbdouroihamaneH.^1^Said AliO.^1^El MessoussiS.^1^KessaR.^2^LahrouniA.^1^BelghaziT.^2^El MerchtS.^2^ChakibE.-H.^2^1Université Cadi Ayyad, Faculté des Sciences Semlalia Marrakech2Centre Régional de Recherche Forestière, Marrakech

### Contribution of Argan Trees in Carbon Sequestration in the Face of Global Warming

Said AliO.^1^AbdouroihamaneH.^1^El MessoussiS.^1^BelghaziT.^2^LahrouniA.^1^El MerchtS.^2^ChakibE.-H.^2^kessaR.^2^1Université Cadi Ayyad, Faculté des Sciences Semlalia Marrakech2Centre Régional de Recherche Forestière, Marrakech

### Demonstration of the Effect of the Argan Tree on the Physicochemical and Microbiological Quality of Camelin Milk

MerchaI.^1^,^2^*LakramN.^2^*ZkhiriF.^1^*El MaadoudiE. H.^2^*BenaliA.^2^*KabbourR.^2^*1Laboratoire de Virologie, Microbiologie & Qualité/Eco-toxicologie & Biodiversité, Université Hassan II, Faculté des sciences et techniques de Mohammedia, BP 146 Mohammadia 20650 Maroc2INRA, CRRA-Rabat, P.O. Box 6570, Institut Rabat, 10101, Rabat Maroc*Correspondence: ikram.mercha@gmail.com

### Effect of the Incorporation of Argan Tree By-Products on Dairy Performance of Sardi Breed

MoutikS.^1^*LakramN.^2^BendaouM.^2^EssafiN.^1^El HousniA.^2^1Laboratoire de chimie organique et étude physico-chimique, Ecole Normale Supérieure, Université Mohammed V, Avenue Mohamed Ben Hassan El OuazzaniaB.P5118 Rabat, Maroc2Laboratoire de technologie alimentaire URPAF, Institut National de la Recherche Agronomique, Rabat, Maroc*Correspondence: moutik.sanaa@gmail.com

### Alcalin Detoxification of the Argan Tree Meal and Impact on Its Nutritional Quality

LakramN.^1^,^2^*MoutikS.^2^EnnahliY.^2^ZouhairF. Z.^2^NaciriM.^1^BendaouM.^2^El MaadoudiE. H.^2^KabbourR.^2^El HousniA.^2^1Laboratoire de Zoologie et Biologie Générale, Université Mohammed ᴠ, Faculté Des Sciences Rabat, Avenu Iben Btouta B.P. 1014, Rabat Maroc2INRA, CRRA-Rabat, P.O. Box 6570, Institut Rabat 10101, Rabat Maroc*Correspondence: nazha.lakram@gmail.com

### Impact of the Origin of the Argan Tree Fruit on the Composition and Quality of Edible Oil

EnnahliY.^1^MehjoubiS.^1^ZouhairF.Z.^1^,^2^BenaliA.^1^KebbourR.^1^1Laboratory of Food Technology URPAF, National Institute for Agricultural Research, Rabat, Morocco2Laboratory of Agroresources and Process Engineering, Faculty of Sciences, University Ibn Tofail, B, P14000 Kenitra, Morocco

### Measurement of Antioxidant and Anti-Glycation Capacities of Argania spinosa Oil

AmakranA.*HamoudaneM.AbidarS.OudainaW.OudainaW.FathiaC.NhiriM.Laboratoire de Biochimie et Génétique Moléculaire, Faculté des Sciences et Techniques, Université Abdelmalek Essaâdi Tanger*Correspondence: amakran_amina@hotmail.com

### Argan Oil and Thrombosis: Experimental Study in Animals

MekhfiH.*BelmekkiF.BnouhamM.ZiyyatA.LegssyerA.AzizM.Laboratoire de Physiologie, Génétique et Ethnopharmacologie, Université Mohammed 1er, Faculté des sciences, Oujda, Maroc*Correspondence: hmekhfi@yahoo.fr

### Effect of Argan Oil on Cardiovascular and Hepatic Complications of Metabolic Syndrome

MouhibM.^1^BenhilalA.^2^OuazaneR.^2^El MessalM.^3^HabbalR.^2^AdlouniA.^1^*1Unité de Pathologie Métabolique et Immunitaire, Laboratoire de Biologie et Sante, Faculté des Sciences Ben Msik, Université Hassan II, Casablanca2Département de Cardiologie, CHU Ibn Rochd, Casablanca3Laboratoire de Biochimie, Faculté des sciences Ain Chock, Casablanca, Université Hassan II, Casablanca*Correspondence: adlounia@yahoo.fr

### Molecular Bases of the Lightening Effect of Argan Oil and of Some by-Products of the Argan Tree on the Skin

BourhimT.^1^MakbalR.^1^VillarealM.^2^HafidiA.^1^GadhiC.^1^*IsodaH.^2^1Faculty of Sciences Semlalia, Cadi Ayyad University, Avenue Prince Moulay Abdellah, BP 2390, 40000 Marrakesh, Morocco2Faculty of Life and Environmental Sciences, University of Tsukuba, Tennodai 1-1-1, Tsukuba City, Ibaraki 305-8572, Japan*Correspondence: dgadhi@uca.ac.ma; isoda.hiroko.ga@u.tsukuba.ac.jp;

### Acute Alcoholism and Anxiety-Depressive Disorders in the Wistar Rat: Neuroprotective Role of Argan Oil

HichamEL Mostafi^1^,^2^TarikTouil^1^,^2^BilalKaarouche^1^AbderrahimLaaziz^1^AliOuichou^1^AboubakerElhessni^1^AbelhalimMesfioui^1^1Laboratoire Génétique, Neuroendocrinologie et Biotechnologie -Faculté des Sciences, Ibn Tofail Université, Kenitra. Maroc2Institut Supérieur des Professions Infirmières et Techniques de Sante de Rabat, Maroc

### Preventive Effect of a Nutritional Supplementation with Argan Oil against Emotional and Mnemonic Disorders Related to Early and Chronic Stress

ZouakiM.BousalhamR.BenazzouzB.OuichouA.El HessniA.LaazizA.AkhouayriO.MesfiouiA.Laboratoire de Génétique, Neuroendocrinologie et Biotechnologie, Faculté des Sciences, Université Ibn Tofail, Kénitra, Maroc

### Argon oil Anticonvulsant Roles against Epileptic-Evil State Development in the Pilocarpine Model of Temporal Lobe Epilepsy in the Wistar Rat

BahbitiYoussefAmmouriHammouBerkiksInssafEl HessniAboubakerOuichouAliNakacheRedouanChakitMiloudBikjdaoueneLeilaMesfiouiAbdelhalemLaboratoire de Génétique, Neuroendocrinologie et Biotechnologies, Faculté des Sciences, Université Ibn Tofail Kenitra

## 4. Poster Presentations Session: Argan.

### Does Argan Dietary Uptake Reverses Memory Impairment Processes in Wistar Rats?

BoulbaroudS.^a^AzzaouiF-Z.^b^Mamadou CireD.^c^NajimiM.^d^AhamiA.O.T.^c^aPolydisciplinary Faculty, Sultan Moulay Slimane University-Beni Mellal-MoroccobLaboratory of Biology and Health, URAC 34, Faculty of Science Ben M’sik, Hassan II University, Casablanca-MoroccocUnit of Neurosciences and applied Nutrition, Faculty of Sciences, Ibn Tofail University, Kenitra-MoroccodFaculty of Sciences and Technology, Beni Mellal-Morocco

Argania spinosa (Argan) and Baillonella toxisperma (Moabi) belong to the class of Sapotaceae. The entire tree is used in African traditional medicine, especially in the Moroccan and Cameroonian pharmacopeia. The fruits seeds of these trees are consumed as food or used as cosmetic products. Their oils are rich in Long chain polyunsaturated fatty acids (LC-PUFA), this component plays essential roles in brain functions, including brain plasticity and memory processes. In this context, the purpose of this work was to study the effect of PUFA supplementation on short and long term memory processes. The Acute oral toxicity studies were carried out according to the Organization for Economic Co-operation and Development (OECD) guideline No. AOT-425 and the Food and Drug Administration (FDA) to evaluate toxicity and to determine the minimum lethal dose of the used oils. A total of 20 adult male rats, weighed (170 ± 6) g, were randomly divided into three groups: (1) T: control groups, (2) Moa 750: groups received Moabi oil at 750 mg/kg, (3) Arg 750: groups received Argan oil at 750 mg/kg. The animals were kept under treatment for 1 month. An object recognition task was used to evaluate the short and long term memory. The Time spent exploring each object was recorded. Intact memory for the familiar object was demonstrated if the rat exhibited a “preference” for the novel object in the choice phase. A preference was indicated if the rat spent more time than chance with the novel object. The results showed that, at a dose level of 2000 mg/kg, neither mortality nor any clinical signs of toxicity were observed. The Moa750 was associated with a significant alteration in short and long term memory tested in memory tasks compared to the control rat, in contrast the administration of Argan oil into rats improved their memory impairment.

**Keywords:** Argan oil; memory impairment; object recognition task; Moabi oil; wistar rat

### Argan Oil and Clove Essential Oil Improves Biochemical and Histological Change by Reducing Oxidative Stress Induced by Hydrogen Peroxide in Wistar Rats

BAKOURMeryemSOULONajouaIMTARAHamadaLYOUSSIBadiaaLaboratory of Physiology-Pharmacology-Environmental Health, Faculty of Sciences Dhar El Mehraz, University Sidi Mohamed Ben Abdallah, Fez, Morocco

Oxidative stress is an imbalance between the generation of reactive oxygen species (ROS) and the antioxidant defenses of the organism, There is a very strong relation between the increase in oxidative stress and the appearance of diseases like cancer and diabetes .This study aims to evaluate the protective effect of argan oil and clove (*Syzygium aromaticum*) essential oil on oxidative stress induced by hydrogen peroxide (H_2_O_2_) in wistar rats, for these reasons the antioxidant content of argan oil and clove essential oil was studied. Rats were randomly divided into six groups (*n* = 6), three groups were kept as control and received (10 mL/kg b.w) of distilled water or clove essential oil prepared in argan oil with the concentration of (100 mg/kg b.w), or argan oil (10 mL/kg b.w), the other three groups received daily by gavage 1% of hydrogen peroxide (H_2_O_2_) (10 mL/kg b.w) and (10 mL/kg b.w) of distilled water or clove essential oil prepared in argan oil with the concentration of (100 mg/kg b.w), or argan oil (10 mL/kg b.w). At the end of the study Biochemical parameters and histological studies were performed. Results indicated that the group which is treated with argan oil supplemented with clove essential oil is the most protected group against oxidative stress induced by hydrogen peroxide.

**Keywords:** hydrogen peroxide; oxidative stress; argan oil; clove essential oil

### Silicon Effect on the Improvement of Black Cumin (Nigella Sativa l.) TOTAL Phenols Content and Other Parameters under Salinity Conditions

FahimiJ.^1^,^2^BouzoubaâZ.^2^AchemchemF.^1^SaffajN.^1^MimouniA.^2^MamouniR.^1^1Team of Materials, Catalysis and Natural Resources Development, Department of Chemistry, Ibn-Zohr University, FSA. Agadir2Agrophysiology & Post Harvest Laboratory Natural Resources and Local Product Research Unity (UR RN & PDT) INRA-CRRA-Agadir

The silicon application is considered as an alternative approach to alleviate salinity stress and to improve yield and quality in plants. Thus, an experiment was conducted with Black cumin (*Nigella sativa* L.) cultivar seeding in pots, under semi-controlled greenhouse conditions in order to determine the silicon effect on total phenols, leaf proline, relative water content (RWC), and dry matter accumulation. The experimental design was an aleatoire complete block (ACB) with three repetitions. Three salinity levels of water irrigation (0, 50 and 100 Mm) were applied. The Results of one way ANOVA test showed a significant increase of leaf proline content, Na+ concentration and a significant decrease of dry matter and RWC in salt conditions, whereas, the application of silicon increased the total phenols content as well as the biometric plants parameters in the same salt conditions. As a conclusion, application of silicon reduced the adverse effect of salinity of black cumin and gives more quality of the product.

**Keywords:** silicon; *Nigella sativa* (L). salinity; total phenol contents; quality

### Nanoparticle-Based Assay for the Detection of Virgin Argan Oil Adulteration and Its Rapid Quality Evaluation

SalghiR.^1^ZougaghM.^2^DhairS.^1^RiosA.^2^1Equipe Chimie Appliquée & encvironnement, Ecole Nationale des sciences Appliquées d’Agadir, B.P: 1136 Maroc2Department of Analytical Chemistry and Food Technology, University of Castilla—La Mancha, 13004 Ciudad Real, Spain

Argan oil is produced from the fruits of the argan (Argania spinosa), a species of tree endemic to south-western Morocco and protected by UNESCO. The resulting oil is slightly darker than olive oil and has a reddish tinge. It is well known for its cosmetic, pharmaceutical and nutritional virtues. Of particular impor- tance are its rich aroma and nutty flavour, which make this oil an exotic ingredient around the world. Recently, it was shown that the geographical origin of the argan fruit and the extraction method used to produce the oil have a considerable influence on its physicochemical composition and characteristics. A new method, based on the formation of gold nanoparticles (AuNPs) and spectrophotometric analysis, is proposed to determine total phenolic acids in virgin argan oil samples. These compounds have reducibility due to the presence of the phenol group in their molecular structure, and a redox reaction occurs in the presence of HAuCl4. The formation of AuNPs as a result of the redox reaction leading to colour changes can be visually observed, resulting in strong light signals that show absorption at 555 nm. As ferulic acid represents more than 95% of the total phenolic acid content of virgin argan oil, this compound was used as an adulteration marker to carry out the screening of samples for the evaluation of the authenticity of virgin argan oils. The analytical features of this screening method also allowed a low precision quantization of the quality of the product. Then, a reference HPLC-DAD/FD method was used to confirm the potential adulterated samples, as well as to provide a detailed quantitative analysis of the most representative phenolic compounds in the samples. The overall screening-confirmation strategy was validated by analysing pure virgin argan oil samples and argan oil samples adulterated with other commercial vege- table oils, demonstrating the reliability of the results. This approach is characterised by its simplicity, low cost, rapid information and responded to practical laboratories needs.

**Keywords:** gold nanoparticles; liquid chromatography; virgin argan oil; adulteration

### Fine-Tuning of Argan Oil Concentrations for a Better Liquid Storage of Boujaâd Ram Semen

El AmiriBouchraAllaiLarbiBen MoulaAnassBadiAbdelmoughitEssamadiAbdelkhalidNasserBoubkerDruartXavier^a^ INRA-Centre Régional de la Recherche Agronomique de Settat, BP589, Settat, Morocco^b^ Laboratoire de Biochimie et Neurosciences, Faculté des Sciences et Techniques, Université Hassan 1, BP 577, 26000 Settat, Morocco^c^ INRA, UMR 85 Physiologie de la Reproduction et des Comportements, F-37380 Nouzilly, France

Argan seed oil (ARO) is harvest from *Argania spinosa (L.)* known as an endemic tree to Morocco and known worldwide for its oil. This oil is extremely rich in unsaturated fatty acids, tocopherols (alpha, beta, gamma and delta) as well as in phenolic acids (vanillic acid, ferulic acid and syringic acid. With caracteristics). The two last components could give to this oil a beneficial role in liquid storage of ram semen. Thus, the aim of this study was to investigate effects of different concentration of argan oil (ARO) on spermatologic parameters, lipid peroxidation and DNA fragmentation during ram semen liquid storage. Also, effects of extenders and temperature on the same parameters were assessed. Semen samples were collected from Boujaâd rams, extended with Tris egg yolk or skim milk extenders without (control) or supplemented with different concentrations of ARO (1, 2, 5 and 10% *v*/*v*) at a final concentration of 0.8 × 10^9^ sperm/mL and stored at 5 °C or 15 °C. The sperm quality assessments were performed at different intervals during storage (0, 8, 24 and 48 h). Sperm progressive motility started to decrease after 8 h of storage in all temperatures—extenders combinations and dropped steadily during the 8–48 h interval. However, sperm viability, progressive motility and membrane integrity were markedly higher in ARO groups (especially in 1% in Tris and 5% in skim milk) until 24 h and 48 h storage at both temperatures compared to controls. The argan oil also decreased the level of spontaneous and induced malondialdehyde (MDA) and the sperm DNA fragmentation until 48 h of storage. In conclusion, argan oil is able to maintain a better quality of ram semen during liquid storage. Moreover, depending on the extender and storage temperature the usage of precise concentrations of argan oil may last storage period. Future studies should aim at determining the exact component of argan oil which is responsible of all improvements recorded in this study.

**Keywords:** Boujaâd rams; argan oil; liquid storage; sperm parameter

## The following abstracts were submitted in French and only displayed by Title, Authors and Affiliations.

### Effect of Argan Oil on the Physicochemical and Microbiological Quality of Milk Camelin

MerchaI.^1^,^2^*LakramN.^2^ZkhiriF.^1^El MaadoudiE. H.^2^BenaliA.^2^KabbourR.^2^1Laboratoire de Virologie, Microbiologie & Qualité/Eco-toxicologie & Biodiversité, Université Hassan II, Faculté des sciences et techniques de Mohammedia, BP 146 Mohammadia 20650 Maroc2INRA, CRRA-Rabat, P.O. Box 6570, Institut Rabat, 10101, Rabat Maroc*Correspondence: ikram.mercha@gmail.com

### Study of the Antidiabetic Effect of Argan Oil by Molecular Interaction

BouchentoufSalimLaboratoire des Substances Naturelles et Bioactives, Université de Tlemcen, Algérie; bouchentouf.salim@yahoo.fr

### Effects of Argan Oil on Blood Pressure and Oxidative Stress in Chronically Glucose-Fed Rats

El MidaouiA.^1^,^2^Filali-ZegroutiY.^1^El HaidaniA.^1^CoutureR.^2^1Équipe de Recherche: “Biologie, Environnement & Santé”, Département de Biologie; FSTE—Université Moulay Ismaïl; Errachidia—Maroc2Département de Physiologie Moléculaire et Intégrative, Faculté de Médecine, Université de Montréal, Montréal, Canada

### Effects of Argan Oil on Hyperglycemia, Hyperinsulinemia, Insulin Resistance, and NADPH Oxidase Activity in Chronically Glucose-Treated Rats

El MidaouiA.^1^,^2^Filali-ZegroutiY.^1^El HaidaniA.^1^CoutureR.^2^1Équipe de Recherche: “Biologie, Environnement & Santé”, Département de Biologie; FSTE—Université Moulay Ismaïl; Errachidia—Maroc2Département de Physiologie Moléculaire et Intégrative, Faculté de Médecine, Université de Montréal, Montréal, Canada

### Molecular Bases of the Lightening Effect of Argan Oil and Some by-Products of The Argan Tree on the Skin

BourhimThouria^1^MakbalRachida^1^VillarealMyra^2^*HafidiAbdellatif^1^GadhiChemseddoha^1^IsodaHiroko^2^*1Faculty of Sciences Semlalia, Cadi Ayyad University, Avenue Prince Moulay Abdellah, B P2390, 40000 Marrakesh, Morocco2Faculty of Life and Environmental Sciences, University of Tsukuba, Tennodai 1-1-1, Tsukuba City, Ibaraki 305-8572, Japan*Correspondence: dgadhi@uca.ac.ma (M.V.); isoda.hiroko.ga@u.tsukuba.ac.jp (H.I.)

### Study Swelling of Samples of Argan Nut Broye Glue with Polypropylene and Water

BabtyFouadDerouicheAbdelaliEl FassiSaadMordaneSoumiaBettachyAminaLaboratoire de Physique des Polymères et Phénomènes Critiques. Université Hassan II-Casablanca, Faculté des Sciences Ben M’Sik, Casablanca

### Alcalin Detoxification of the Argan Tree Meal and its Impact on its Nutritional Quality

LakramN.^1^,^2^*MoutikS.^2^EnnahliY.^2^ZouhairF.Z.^2^NaciriM.^1^BendaouM.^2^El MaadoudiE. H.^2^KabbourR.^2^El HousniA.^2^1Laboratoire de Zoologie et Biologie Générale, Université Mohammed ᴠ, Faculté Des Sciences Rabat, Avenu Iben Btouta B.P. 1014, Rabat Maroc2INRA, CRRA-Rabat, P.O. Box 6570, Institut Rabat, 10101, Rabat Maroc*Correspondence: nazha.lakram@gmail.com

### Evidence of the Effect of the Argan Tree on the Physicochemical and Microbiological Quality of Camelin Milk

MerchaI.^1^,^2^*LakramN.^2^ZkhiriF.^1^El MaadoudiE. H.^2^BenaliA.^2^KabbourR.^2^1Laboratoire de Virologie, Microbiologie & Qualité/Eco-toxicologie & Biodiversité, Université Hassan II, Faculté des sciences et techniques de Mohammedia, BP 146 Mohammadia 20650 Maroc2INRA, CRRA-Rabat, P.O. Box 6570, Institut Rabat 10101, Rabat Maroc*Correspondence: ikram.mercha@gmail.com

### The Impact of the Origin of the Argan Tree Fruit on the Composition and the Quality of Edible Oil

EnnahliY.^1^MehjoubiS.^1^ZouhairF.Z.^1^,^2^BenaliA.^1^KebbourR.^1^1Laboratory of Food Technology URPAF, National Institute for Agricultural Research, Rabat, Morocco2Laboratory of Agroresources and Process Engineering, Faculty of Sciences, University Ibn Tofail, B, P14000 Kenitra, Morocco

## Special Session “Benefits of Natural Plant Extracts on Human Health”.

### Antidiabetic Effect of Anabasis Aretioides in Streptozotocin-Induced Diabetic Rats 

FaridO.*HebiM.AjebliM.El BouhaliB.HajjiL.El HaidaniA.EddouksM.Faculty of Sciences and Techniques Errachidia, Moulay Ismail University, BP 509, Boutalamine, 52000 Errachidia, Morocco*Correspondence: faridomar@gmail.com

In this study the effect of *Anabasis aretioides* (A. aretioides) aerial part aqueous extract (A.P.A.E) at a dose of 5 mg/kg body weight on blood glucose levels and plasma lipid profile was investigated in normal and streptozotocin (STZ) diabetic rats. A preliminary screening for various bioactive constituents was realized and the antioxidant potential of the aqueous extract was also demonstrated. The histopathological changes in liver and pancreas have been evaluated both in normal and STZ diabetic rats. The ability of A. aretioides aqueous extract to improve glucose tolerance in normal rats was also determined. Furthermore, the relative organs weight (R.O.W) of liver, kidney, pancreas and brown adipose tissue were given. 

In normal rats, a single administration of the A.P.A.E (5 mg/kg) has not shown a significant reduction in blood glucose levels. However, a significant reduction (*p* < 0.0001) was observed in diabetic rats. Repeated oral administration of A. aretioides exerted a significant reduction in blood glucose levels both in normal (*p* < 0.05) and STZ diabetics (*p* < 0.0001) rats. The blood glucose lowering activity of A.P.A.E was comparable to vanadate treatment at a dose used. The oral glucose tolerance test demonstrated the ability of the aqueous extract of A.P.A.E (5 mg/kg) to improve the increase on blood glucose levels in normal treated rats. In this investigation no significant changes in body weight in normal and STZ rats were shown. According to the DPPH (1-1-diphenyl 2-picryl hydrazyl) radical scavenging activity, the aqueous extract has shown a not negligible antioxidant activity.

**Keywords:** Anabasis aretioides; aqueous extract; streptozotocin; lipid profiles; histopathological changes.

* This work was supported by the CNRST under the grant number PPR/2015/35.

### Preliminary Phytochemical Screening and Evaluation of Hypolipidemic Activity of the Aerial Part of Aqueous Extract of Tamarix Articulata Vahl

HebiM.HajjiL.El HaidaniA.EL BouhaliB.EddouksM.Faculty of Sciences and Techniques Errachidia, Moulay Ismail University, BP 509, Boutalamine, 52000, Errachidia, Morocco; mohamed.eddouks@laposte.net

The objective of this study was to evaluate the hypolipidemic activity of aqueous extract of *T. articulata* in normal and streptozotocin-induced rats. Phytochemical screening as well as polyphenol and flavonoid contents in aqueous extract of T. articulate were evaluated. 

The plasma concentrations of Total cholesterol, Triglycerides and High-Density Lipoprotein-cholesterol (HDL-c) were measured in STZ-diabetic and normal rats previously treated by aqueous extract of *T. articulata* (5 mg/kg of body weight). Total phenolic content of the aqueous extract was determined by Folin Ciocalteu method. Concerning flavonoids content, the colorimetric AlCl3 method was used. 

A single oral administration in diabetic rats showed a significant increase in the HDL-cholesterol after 6 hours of treatment with *T. articulata* (5 mg/kg). The results demonstrated also that the aqueous extract of *T. articulata* produced a significant decrease in serum total cholesterol on repeated oral administration in streptozotocin diabetic rats (*p* < 0.001). In contrast, plasma HDL-cholesterol levels were increased significantly after 7 days of treatment with *T. articulata* (*p* < 0.001). The total polyphenol and flavonoid contents of *T. articulata* aqueous extract were 102.5 mg EAG/g of extract and 54.83 mg EQ/g of extract, respectively. According to preliminary phytochemical screening of aerial part of *T. articulata* several classes of chemicals have been found such as polyphenols, flavonoids, tannins, cyanidins (flavones, catecols), mucilage, sesquiterpenes, terpenoids and carbohydrates. 

We conclude that *T. articulata* aqueous extract exhibits hypolipidemic potential. 

**Keywords:** hypolipidemic; streptozotocin; total cholesterol; triglycerides; High-Density Lipoprotein-cholesterol (HDL-c); polyphenols; flavonoids.

Funding: This work was supported by CNRST under grant number PPR/2015/35.

### Study of Glucose and Lipid Lowering Activity of Mentha Suaveolens Ehrh. In Normal and Streptozotocin-Induced Diabetic Rats

AjebliM.*EL HaidaniA.HajjiL.EL BouhaliB.EddouksM.Faculty of Sciences and Techniques, Moulay Ismail University, BP 509, Boutalamine, 52000 Errachidia, Morocco*Correspondence: mohammed.ajebli@gmail.com

*Mentha suaveolens* Ehrh. is an aromatic and medicinal plant from Lamiacea family. This plant has many food and medicinal uses including diabetes management. The present work aimed to evaluate the effect of the aqueous extract of aerial part of *Mentha suaveolens* Ehrh (AEAPMS) on blood glucose levels and the plasma lipid profile. Blood glucose and plasma lipid levels were followed after both single and repeated (15 days of treatment) AEAPMS (20 mg/Kg) oral administration in normal and streptozotocin-induced diabetic rats. 

Furthermore, preliminary phytochemical screening, quantification of phenolic and flavonoid contents as well as histopathological examination of pancreas and liver were carried out. The results showed that AEAPMS exhibited a significant glucose and lipid (Total cholesterol and triglycerides) lowering activity in both normal and diabetic rats. However, no significant effect was observed on serum lipoproteins (HDL and LDL). Moreover, AEAPMS seems to act positively on histopathological tissues of liver and pancreas. The phytochemical analysis of AEAPMS showed that the main phytochemical constituents of *Mentha suaveolens* are alkaloids, polyphenols, flavonoids, cyanidins, tannins, glucosides, saponins, quinones, anthraquinones, mucilage, sterols, sesquiterpenes, reducing sugars, carbohydrates and terpenoids. On another hand, a potential in vitro antioxidant of AEAPMS has been shown. 

In conclusion, the study demonstrates that AEAPMS possesses a beneficial effect on glucose and lipid metabolism. This effect might be mediated through the amelioration of the liver and pancreas function. 

**Keywords:** Antidiabetic; lipid profile; mentha suaveolens Ehrh; histopathology; antioxidant activity

* This work was supported by the CNRST under the grant number PPR/2015/35.

### Anti-inflammatory properties and phenolic profile of six moroccan date fruit (phoenix dactylifera) varieties

BOUHLALIEimad dine Tariq^a^^b^EL HILALYJaouad^a^^c^^d^RAMCHOUNMhamed^e^^b^HMIDANIAbdelbassat^a^^b^EL MIDAOUIAdil^a^ALEMChakib^b^AMAROUCHMohamed‑Yassine^a^^c^FILALI-ZEGZOUTIYounes^a^aBiology, Environment & Health Team, Faculty of Sciences and Techniques ErrachidiabBiochemistry of Natural Product Team, Faculty of Sciences and Techniques ErrachidiacMaterials, Natural Substances, Environment and Modeling Laboratory, Multidisciplinary Faculty of TazadRegional institute of Education and Training Careers, FezeFaculty of medicine and pharmacy, Beni Mellal

Date fruit (*Phoenix dactylifera*) is traditionally used in Morocco to treat inflammatory diseases including rheumatoid arthritis, but this has not yet been studied at the scientific level. In this study, phenolic profile and anti-inflammatory activity of six date fruit varieties were assessed. The results showed that Gallic, Ferulic, Caffeic acids and Rutin were the most dominant among analysed polyphenolic compounds. Regarding the anti-inflammatory activity Bousrdoun possessed the highest nitric oxide scavenging ability (IC_50_ = 531.34 μg/mL) and induced the highest protein denaturation inhibition (IC_50_ = 408.64 μg/mL) as well as the highest membrane stabilizing effect (IC_50_ = 483.61 μg/mL). *Jihl* exhibited the highest reduction of ear edema (74%) and the highest inhibition of paw oedema at 4th hour after carrageenan injection was found in *Jihl* and *Bousrdoun* with an average of paw swilling (40.35%). In conclusion, these results support the use of date fruit and its main compounds for their anti-inflammatory properties and antioxidant activity. The difference observed between analysed date fruit verities regarding their anti-inflammatory activiy may be due in part to the variations in the phenolic profile and total phenols and flavonoids content. 

**Keywords:** Date fruit; phenolic profile; Anti-inflammatory activity

### Functional Composition and Biological Activities of Six Moroccan Date Fruit Varieties Grown in Southeastern Morocco

BOUHLALIE.D.T.^a^^b^RamchounM.^b^^c^SellamK.^a^BammouM.^a^HmidaniA.^a^KhouyaT.^b^BenlyasM.^a^AmarouchM.Y.^a^^d^EL MidaouiA.^a^AlemC.^b^FILALI-ZegzoutiY.^a^aBiology, Environment and Health Team, Faculty of sciences and Techniques Errachidia, MoroccobBiochemistry of Natural Product Team, Faculty of sciences and Techniques Errachidia, MoroccocFaculty of Medicine and Pharmacy Beni Mellal, Morocco;dMaterials, Natural Substances, Environment and Modeling Laboratory, Multidisciplinary Faculty of Taza

The aim of this study was to determine the functional composition and biological activities of some date fruit varieties that are unknown to the general public, but highly appreciated by the local populations of oases these varieties are locally known as *Boufgous, Bouskri, Bousrdon, Bousthammi, Jihl* and *Majhoul*. Sucrose was the main sugar in *Bouskri* unlike other varieties which contain fructose and glucose in high amount. *Bouskri* contains the highest amount of pyridoxine and *Majhoul* possesses the highest amount niacin and riboflavin. Among analyzed cultivars, *Bousthammi* contains the highest amount of magnesium and manganese and has iron seven times more than *Jihl* cultivars, which contains a high amount of calcium and copper. The highest amount of zinc was found in *Najda* while the highest amount of potassium was detected in *Bouskri*. Significant difference in antioxidant activity was observed between analyzed varieties. The highest antioxidant activity was observed in both *Bousrdoun* and *Jihl* which contained the highest amount of phenolic and flavonoids content. Gallic, Ferulic, Caffeic acids and Rutin were the most dominant among analysed polyphenolic compounds and the highest amount of these compounds were observed in *Jihl* and *Bousrdoun.* Regarding the anti-inflammatory activity and based on the results of three in vitro assays and two in vivo assays, *Bousrdoun* and *Jihl* exhibited the highest anti-inflammatory effect. Moderate antibacterial activity was observed in all studied varieties except Bouskri and Majhoul which didn’t show no antibacterial effect against the six bacterial strains. *Bousrdon* and *Jihl* were found to be more potent against these bacterial strains. These differences in chemical composition and biological activity suggest that date fruit is an extensive domain hence the size should not be the only character that should be used to choose the variety but also its composition and their biological activities. 

**Keywords:** antioxidant; anti-inflammatory; antibacterial; date fruit functional composition 

### Ethnobotany Study of Medicinal Plants Used in the Treatment of Certain Diseases in the City of Errachdidia

El-HaidaniA.^1^SalhilS.^1^BouhlaliE.-T.^1^El AchhabY.^2^El MidaouiA.^1^HalouiZ.^3^Filali-ZegzoutiY.^1^1Biology department, Sciences and Technics Faculty of Errachidia, Errachidia, Morocco2Regional Center for Careers Education and Training of Fez-Meknes, Taza, Morocco3Lab. of Environment & Health, Kénitra, Morocco

A large number of aromatic and medicinal plants have very interesting biological properties, which find applications in various fields such as medicine, pharmacy, cosmetology and agriculture. In order to know the medicinal plants traditionally by the population of the city of Errachidia, an ethnobotanical study was carried out in this city. The study of the medicinal flora made it possible to inventory 48 species belonging to 23 families. The ethnobotanical survey carried out by means of a questionnaire, made it possible to collect a certain number of information. Foliage is the most widely used part of medicinal plants. The method of preparation of the majority of remedies is the decoction. In all the diseases treated, digestive diseases represent the most cited diseases.

**Keywords:** medicinal plants; digestive diseases; City of Errachidia

### Screening of Biological Activity of Polyphenol-Rich Extracts from South East Moroccan Thyme Varieties

RamchounM.^1^,^3^KhouyaT.^2^HmidaniA.^2^HarnafiH.^3^SellamK.^2^BouhlaliE.-T.^2^ilali ZegzoutiY.^2^AlemF. C.^2^BenlyasM.^2^NazihH.^4^OuguerramK.^5^AmraniS.^3^1Sultan Moulay Slimane University, 23000 Beni Mellal, Morocco2Biochemistry of Naturel product Equipe, Department of Biology, Faculty of Sciences and Technology 52000 Errachidia, University Moulay Ismail, Morocco3Laboratory of Biochemistry, Department of Biology, Faculty of Sciences, 60000 Oujda, Morocco4MMS2160 Laboratory of Biochemistry, Faculty of Pharmacy, University of Nantes, France5Research Center in Humain Nutrition, INSERM U 915, CHU of Nantes, France

Cardiovascular disease is the most frequent cause of death in industrialized countries and hyperlipidemia represents a major risk factor for the premature development of atherosclerosis and its vascular complications. Despite, the availability of highly effective treatments, morbidity and mortality from atherosclerotic vascular diseases remain substantial. Therefore, novel therapies to improve cardiovascular outcomes are needed. Thus, natural compounds might represent an alternative therapeutic approach. Indeed, several epidemiological and experimental studies have shown that intake of a number of polyphenols from medicinal plants, which exert protective effects against oxidative stress, is inversely correlated with atherosclerosis development and cardiovascular events.

The genus Thymus L. (Lamiaceae), aromatic plants of the Mediterranean flora, economically important due to their use in folk medicine for their numerous medicinal and aromatic properties, have been reported to possess various biological effects including antioxidant and antimicrobial activities. *Thymus satureioides* (*T. satureioides*), *Thymus atlanticus* (*T. atlanticus*) and *Thymus zygis* (*T. zygis*) are an endemic species of Morocco used in the folk medicine in form of infuse and decoctions to treat whooping cough, bronchitis and rheumatism and, generally, for its anti-inflammatory properties after topical or oral administration. Many works showed that some Thymus species possess anti-inflammatory activity but to our knowledge, no study has investigated the effect of these plants on the coagulation and hypolipemiant activity.

In this study, we assessed the hypolipemic, anti-inflammatory, coagulation and antioxidant effects of polyphenol-rich extracts obtained from endemic Moroccan thyme varieties (Lamiaceae) collected in the Errachidia area (south east of Morocco).

**Keywords:** Thymus; polyphenol; antioxidant activity; hypolipemic activity; anti-inflammatory activity; anti-coagulation activity; cardiovascular disease

### Evaluation of Medicago Sativa Growth Inoculated with Rhizobial Strain Rhol1 and/or Arbuscular-Mycorrhizal (AM) Fungi under Stress Conditions

Ben LaouaneR.*1*2*3MeddichA.*1FaghireM.*3OufdouK.*2BechtaouiN.*2El AmeranyF.Ait El MokhtarM.*1WahbiS.*1*1LLaboratoire de Biotechnologie et Physiologie Végétale FSSM-Université Cadi Ayyad. Marrakech*2Laboratoire de Biologie et Biotechnologie des Microorganismes FSSM-Université Cadi Ayyad*3Laboratoire de Biotechnologie Végétale Faculté des Sciences Université Ibn Zohr Agadir

Alfalfa *(Medicago sativa)*, is a perennial flowering plant in the family Fabaceae. It is cultivated as an important forage crop in many countries around the world. This plant has been used as a common ingredient in south-west Moroccan (Tinghir) and South Indian cuisine. Alfalfa is rich in many essential vitamins and minerals, including A, D, E, K, and even the full family of B vitamins; biotin, calcium, folic acid, iron, magnesium, potassium …, as well as being very high in protein. This herb is believed to have a direct connection to lowering cholesterol. It is very good at detoxifying and better purifying the blood. It has also beneficial healing properties against bad breath, sore or achy joints, imbalanced skin conditions, and it even increases immune system functionality. This herb acts as an alternative to over the counter pain medicines for headaches or migraines. As a result, consuming alfalfa on a routine basis has an abundance of positive health results. Medicago sativa constitutes the first forage crop in Mediterranean area In Morocco, this crop occupies over 22% of the total area devoted to forage crops. It strongly contributes to socio-economic development of local families. However, water and soil salinity recorded in many world regions is a major environmental factor limiting plant growth and productivity and constitutes an important constraint to alfalfa. In this context, the present study aimed to evaluate the salinity tolerance of Midecago sativa inoculated with Rhizobial strain RhOL1 and/or arbuscular-mycorrhizal (AM) fungi (autochtonous mycorhizal).

**Keywords:** Medicago sativa; stress conditions; arbuscular-mycorrhizal; Rhizobial strain RhOL1; growth

### Does Argan Dietary Uptake Reverses Memory Impairment Processes in Wistar Rats?

BoulbaroudS.^a^AzzaouiF-Z.^b^Mamadou CireD.^c^NajimiM.^d^AhamiA.O.T.^c^aPolydisciplinary Faculty, Sultan Moulay Slimane University-Beni Mellal-MoroccobLaboratory of Biology and Health, URAC 34, Faculty of Science Ben M’sik, Hassan II University, Casablanca-MoroccocUnit of Neurosciences and applied Nutrition, Faculty of Sciences, Ibn Tofail University, Kenitra-MoroccodFaculty of Sciences and Technology, Beni Mellal-Morocco

Argania spinosa (Argan) and Baillonella toxisperma (Moabi) belong to the class of Sapotaceae. The entire tree is used in African traditional medicine, especially in the Moroccan and Cameroonian pharmacopeia. The fruits seeds of these trees are consumed as food or used as cosmetic products. Their oils are rich in Long chain polyunsaturated fatty acids (LC-PUFA), this component plays essential roles in brain functions, including brain plasticity and memory processes. In this context, the purpose of this work was to study the effect of PUFA supplementation on short and long term memory processes. The Acute oral toxicity studies were carried out according to the Organization for Economic Co-operation and Development (OECD) guideline No. AOT-425 and the Food and Drug Administration (FDA) to evaluate toxicity and to determine the minimum lethal dose of the used oils. A total of 20 adult male rats, weighed (170 ± 6) g, were randomly divided into three groups: (1) T: control groups, (2) Moa 750: groups received Moabi oil at 750 mg/kg, (3) Arg 750: groups received Argan oil at 750 mg/kg. The animals were kept under treatment for 1 month. An object recognition task was used to evaluate the short and long term memory. The Time spent exploring each object was recorded. Intact memory for the familiar object was demonstrated if the rat exhibited a “preference” for the novel object in the choice phase. A preference was indicated if the rat spent more time than chance with the novel object. The results showed that, at a dose level of 2000 mg/kg, neither mortality nor any clinical signs of toxicity were observed. The Moa750 was associated with a significant alteration in short and long term memory tested in memory tasks compared to the control rat, in contrast the administration of Argan oil into rats improved their memory impairment.

**Keywords:** Argan oil; memory impairment; object recognition task; Moabi oil; wistar rat

### Chemical Characterization and In Vitro Antimcrobial Activity of Carum Carvi Essential Oil

GhouatiYasmine^1^BelaicheTouriya^1^AmechrouqAli^2^BelmalhaSaadia^3^BoukiliMohammed^4^HalouiZoubida^4^ChakirSaid^4^1Ecole Nationale d’Agriculture, Unité de Technologie Alimentaire et de Biochimie BP S/40 Meknès, Maroc2Faculté des Sciences de Meknès, Département de Chimie, BP: 11021, Zitoune, Meknès, Maroc3Ecole Nationale d’Agriculture, Département de Protection des Plantes et de l’Environnement BP S/40 Meknès, Maroc4Faculté des Sciences de Meknès, Département de Biologie, BP: 11021, Zitoune, Meknès, Maroc

Recently, many antibiotics have been ineffective due to the rapid development of microbial resistance wich led to the emrgency of new infection diseases. Therefore, to overcome this problem the use of essential oil is one of the promising strategies.

The present study was firstly undertaken to determine the chemical composition of *Carum carvi* essential oil obtained by hydrodistillation and to evaluate its antimicrobial effect. The chemical composition was analyzed by a GC/MS system and a total of constituents were identified representing about 86.80% of total oil. The antimicrobial activity of *Carum carvi* essential oil against microbial strains was qualitatively and quantitatively assessed by the presence or absence of inhibition, and MIC values. The studied essential oil exhibited an inhibition against the majority of tested bacteria and was found to be more active against Gram-positive bacteria (*Bacillus cereus* and *Staphylococcus aureus*) than Gram-negative ones (*Escherichia coli* and *Salmonella Typhi*).

The results demonstrate that *Carum carvi* essential oil have an antimicrobial effect. These findings are very promising and suggest that essential oil isolated from *Carum carvi* can be considered as new and potential source of natural antimicrobial agents.

**Keywords:** chemical composition; essential oil; Carum carvi; antimicrobial activity; Gram-positive bacteria; Gram-negative bacteria

### Chemical Composition, Antioxidant and Antibacterial Activities of Essential Oil of the Leaves of Tetraclinis Articulate (Vahl) MASTERS from Morocco

SadikiF.Z.^1^*El IdrissiM.^1^HritcuL.^2^CioancaO.^3^TrifanA.^3^SbitiM.^4^1Laboratory of Molecular Chemistry and Natural Substances , Department of Chemistry, Faculty of Sciences of
Meknes, Moulay Ismail University, Bp. 11201, Zitoune, Meknès, Morocco2Department of Biology, Faculty of Biology, Alexandru Ioan Cuza University of Iasi, Bv. Carol I, no. 11, 700505
Iasi, Romania3Department of Pharmacognosy, Faculty of Pharmacy, University of Medicine and Pharmacy “Gr. T. Popa”, Iasi,Str. Universitatii no. 16, 700115 Iasi, Romania4Laboratory of microbiology, Ismail Military Hospital, Meknes, Morocco*Correspondence: fatizahrasad@gmail.com

Medicinal and aromatic plants have been used for a long time in the process of oxidative stress and the fight against infectious diseases. But the discovery of synthetic antioxidants and antibiotics caused the decline of herbal medicine and relegated it to a secondary rank. In effect, the secondary effects of these synthetic products and the appearance of resistant bacteria led us to study the antioxidant and antibacterial activities of the essential oil of leaves of *Tetraclinis articulata (vahl) Masters*. Extraction of the essential oils was carried out by hydrodistillation. The composition of the essential oil was analyzed by GC/MS. We used to evaluate the antioxidant activity of the essential oil the radical ABTS and the radical DPPH. While the antibacterial activity was determinate by paper disc diffusion method and the liquid dilution method. The test microorganisms include Gram-positive bacteria and Gram-negative bacteria which are clinical isolates. The main constituents of the essential oil were Camphor (24.21%), α-Pinene (23.77%), Bornyl Acetate (17.37%) and Borneol (10.32%). The results of the biological tests show that the essential oil of the leaves of Tetraclinis articulata has interesting antibacterial and antioxidant properties. This study confirms scientifically the traditional use of this plant and reveals its interest in the context of exploitation in biotechnology. 

**Keywords:** *Tetraclinis articulata*; antioxidant; antibacterial; essential oil

### Phytochemistry of the Essential Oil of Cymbopogon Nardus Growing in Morocco: Preventive Approach Against Nosocomial Infections

El KamariFatimaTaroqAmalEl AtkiYassineAouamImaneLyoussiBadiaaAbdellaouiAbdelfattahLaboratoire physiologie pharmacologie & santé environnementale lppse, université sidi mohamed ben abdellah, faculté de sciences dhar mahraz, fès, Maroc

This paper describes chemical composition, and antimicrobial activity of *Cymbopogon nardus* citronella essential oil against bacteria responsible for nosocomial infections. Due to the ban of antibiotics, this study was carried out to evaluate the potential of citronella essential oil as alternative to commercial antibiotics use against nosocomial bacteria.

The phytochemical characterization of essential oil was evaluated using gas chromatography-flame ionization detector and gas chromatography-mass spectrometer analysis. Antibacterial activity of the oil was tested against six bacterial strains responsible for nosocomial infections: Pseudomonas aeruginosa, Klebsiella pneumonia, Staphylococcus aureus, Proteus mirabilis, Escherichia coli and Acinetobacter baumannii using disc diffusion method. 

Eight components were identified, the predominant components were Geraniol, trans-citral and cis-citral. The study of the antibacterial power showed an important antibacterial activity of the essential oil tested against all the bacterial strains. 

These results suggest that the essential oil extracted from *Cymbopogon nardus* can be used to clean the environment of reanimation polyvalent and anesthesia service.

### Phytochemical Screening, Quantitative Analysis and Antioxidant Activity of Vitex Agnus Castus. L. (lamiaceae) from South-East of Morocco

AichaHamdouch^1^TaoufikFatima^2^AsdadiAli^1^ChebliBouchra^3^HassaniLalla Mina Idrissi^1^1Laboratory of Plant Biotechnology, Faculty of Science, University Ibn Zohr, Agadir, Morocco2Laboratory of Chemical Engineering, Department of Chemistry, Faculty of Science, University IBN ZOHRAgadir, Morocco3Laboratory of Biology, School of Applied Sciences, University Ibn Zohr Agadir, Morocco

This study was designed to assess the phytochemical screening of leaves and seeds of *Vitex agnus castus* harvested in a marginalized oasis of south-East of Morocco and to evaluate their potential antioxydante activity properties using 2,2 diphenyl-1-picrylehydrazil (DPPH) radical scavenging. The phytochemical screening revealed the presence of alkaloids, terpenes and catechic tannins in the different samples, anthraquinones in seeds and saponins in leaves. Methanol 80% extract of seeds and leaves of *Vitex agnus castus* was obtained and subjected to a quantitative determination of total polyphenols and total flavonoids content. Leaves and seeds showed a high content of total polyphenols; Total flavonoids content was high in leaves Quantification of aglycones in the two parts of *Vitex agnus castus* revealed and important amount of aglycones in leaves. The thin layer chromatography of methanol 80% extracts revealed a wide variety of flavonoids and the presence of kaempferol in the seeds of *Vitex agnus castus.*

**Keywords:** phytochemical screening; total polyphenols content; total flavonoids content; Aglycons content; antioxidant activity; Vitex agnus castus

### In Vitro Antifungal and Antioxidant Activities of Essential Oil, Polar and Apolar Extracts of Pulicaria Mauritanica from South-East of Morocco

HamdouchA.^1^ChebliB.^2^IdrissiL.M.^1^1Faculty of science, University Ibnou Zohr, Agadir, Morocco2School of applied sciences, University Ibnou Zohr Agadir, Morocco

In vitro antifungal and antioxidant activities of essential oil, methanol and ether extracts and anticorrosive activity of essential oil of *Pulicaria mauritanica* (Asteraceae) harvested in the oasis of Tata of south-East of Morocco were investigated. Essential oil was extracted using hydrodistillation method; methanol and ether extracts were obtained using soxhlet apparatus. Essential oil showed a strong inhibition of *Penicillium digitatum* mycelium growth (100% at 700 ppm); ether extract gave 46.212 ± 1.543% of inhibition at 30,000 ppm, while methanol extract exhibited no activity against *P. digitatum*. Methanol extract showed a strong reduction of DPPH (IC_50_ = 0.027 ± 0.004 mg/mL), while essential oil and ether extract exhibited median reduction of DPPH, IC_50_ are successively 0.080 ± 0.005 mg/mL and 0.123 ± 0.0006 mg/mL. Phytochemical investigation of studied organic extracts showed that polyphenolic compounds constitute the major compounds of it. Linear correlation between total polyphenols and total flavonoids contents and antioxidant capacity was obtained; the correlation was positive

**Keywords:** *Pulicaria mauritanica*; antifuangal activity; antioxidant activity; IC_50_; insecticidal activity; phytochemical analyses

### Phytochemical Screening, Quantitative Analysis and Antioxidant Activity of Pulicaria Inuloides (Poiret) DC. (Asteraceae) from South-East of Morocco

HamdouchAicha^1^*AajmiAbderrahman^2^TaoufikFatima^2^ChebliBouchra^3^HassaniLalla Mina Idrissi^1^1Laboratory of plant biotechnology, faculty of science, University Ibn Zohr, Agadir, Morocco2Laboratory of Process Engineering, University Ibn Zohr Agadir, Morocco3Laboratory of Biology, School of Applied Sciences, University Ibn Zohr Agadir, Morocco*Correspondence: aicha.hamdouch@gmail.com

This study was designed to assess the phytochemical screening of leaves of Pulicaria inuloides (Poiret) DC. harvested in a marginalized oasis of south-East of Morocco and to evaluate their potential antioxydante activity properties using 2,2 diphenyl-1-picrylehydrazil (DPPH) radical scavenging. The phytochemical screening revealed the presence of terpenes, catechic tannins, anthraquinones, free quinons, in methanol and petroleum ether extracts of leaves of *P. inuloides*, alkaloids and saponins are present only in methanol extract. Methanol and petroleum extracts of leaves of *P. inuloides* were subjected to a quantitative determination of total polyphenols and total flavonoids content. Methanol extract showed a high content of total polyphenols and total flavonoids content (32.75 ± 0.0066 mg GAE/g dried extract and 11.025 ± 0.003 mg QE/g dried extract respectively). Quantification of aglycones revealed an important content in leaves of *P. inuloides* (0.210 ± 0.023 mg QE/ g dried plant material). Test of antioxidant activity revealed an important scavenging effect of methanol extract compared to petroleum ether extract (IC_50 =_ 0.05 ± 0.01 mg/mL and IC_50_ = 0.14 ± 0.026 mg/mL respectively).

**Keywords:** phytochemical screening; total polyphenols content; total flavonoids content; antioxidant activity; Pulicaria inuloides

### In vitro Investigation of the Antioxidant and Anti-Inflammatory Activities of Three Thymus Species Grown in Southeastern Morocco

hmidaniAbdelbassat^a^^b^bouhlaliEimad dine Tariq^a^^b^khouyaTarik^b^ramchounMhamed^b^^c^filali zegzoutIYounes^a^alemChakib^b^benlyasMohamed^a^aBiology, Environment and Health Team, Faculty of sciences and Techniques Errachidia, MoroccobBiochemistry of Natural Product Team, Faculty of sciences and Techniques Errachidia, MoroccocFaculty of Medicine and Pharmacy Beni Mellal, Morocco

The aim of the present study is to examine the antioxidant and anti-inflammatory activities of three thyme varieties grown in southeastern Morocco. The antioxidant activity was performed using ABTS assay. In vitro anti-inflammatory assay was also studied through the evaluation of membrane stabilization effect, inhibition of protein denaturation and inhibition of protease activity. The result showed that all varieties possess three important antioxidant activities: *Thymus Atlanticus* (IC _50_ = 16.59 ± 0.32 μg/mL), *Thymus Zygis* (IC _50_ = 15.65 ± 0.74 μg/mL), *and Thymus Satureioides* (IC _50_ = 15, 48 ± 0.35 μg/mL). Concerning the anti-inflammatory activity and based on the results of the in vitro assays used in this study, the highest anti-inflammatory effect was depicted in *Thymus atlanticus* followed by *Thymus zygis* and *Thymus satureioides.* The findings of this survey may partly explain the use of those plants in the Moroccan traditional medicine for the treatment of inflammatory diseases.

**Keywords:** antioxidant; anti-inflammatory; *Thymus* *atlanticus*; *Thymus zygis*; *Thymus satureioides*

### Thin Layer Chromatography Fingerprinting of Thymelaea Lythroides Extracts for Pharmacological Screening 

NasriI.^1^El BouhaliB.^3^BouazS.^1^BoughribilS.^2^BenajiB.^1^EddouksM.^3^1Laboratory of Biochemistry, Environment & Agro Alimentary. Team of Pharmacochemistry. URAC 36. Faculty of Sciences and Techniques. University Hassan II, Casablanca2Laboratory of Virology, Microbiology and Quality. Faculty of Sciences and Techniques. University Hassan II, Casablanca3Team of nutritional physiology and endocrine pharmacology, Faculty of Sciences and Technology, University Moulay Ismail, Errachidia. This work was supported by the CNRST under grant number PPR/2015/35

Medicinal plants constitute an important reservoir of active and new biomolecules and wide variety of secondary metabolites. The obtaining of reproducible and reliable extracts ensures the reproducibility of the pharmacological activity. Chromatography fingerprinting is an elegant approach to monitoring the compounds involved.We develop TLC fingerprint to evaluate the chemical stability and identifies biomolecules of the extract before screening pharmacological activity. The TLC fingerprint is constructed from the spots and the area of the compounds by software.


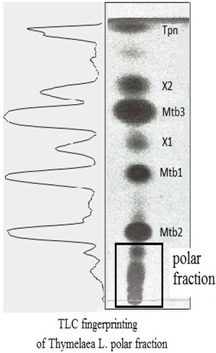


*Thymelaea L.* is an endemic plant known for its ethno-pharmacological use in several pathologies in Morocco. After extraction by several solvents and evaporation, the residues were subjected to TLC on silica gel. The chemical reagents in solution, sprayed on TLC chromatograms, are used to complement the visual observations under sunlight and for a specific revelation. 

In our previous studies differential extraction shows four stable compounds. Three flavonoids (Mtb1, Mtb2 Mtb3) were identified and Tpn is probably a terpene. In this research we are focused on polar fraction for antibacterial and antihypertensive screening. This TLC fingerprint is used in first step for preparative chromatography of polar fraction. The first results show promising pharmacological activity.

TLC fingerprinting localizes easily the secondary metabolites and optimizes the fractions for preparative chromatography. It also provides a simple and fast method to monitor a particular component in the mixture. The demonstration of pharmacological activity of family or single molecule becomes more rational.

**Keywords:** TLC fingerprints; Thymelaea L.; antihypertensive; antibacterial activity

### Phytochemistry of the Essential oil of Melissa Officinalis l. Growing Wild in Morocco

JalalZinebLyoussiBadiaaAbdellaouiAbdelfattahLaboratory of Physiology Pharmacology and Environmental Health, Department of Biology, Faculty of Sciences Dhar Mehraz, University Sidi Mohamed Ben Abdellah, B.P. 1796, Atlas, Fez, Morocco

To determine the phytochemical characterization and antibacterial activity of *Melissa officinalis* essential oil against bacteria responsible for nosocomial infections.

The phytochemical characterization of essential oil was evaluated using gaschromatography-flame ionization detector and gas chromatography-mass spectrometeranalysis. Antibacterial activity of the oil was tested against four bacterial strainsresponsible for nosocomial infections: *Pseudomonas aeruginosa, Klebsiella pneumonia, Staphylococcus aureus and Citrobacter koseri* using disc diffusion method.

Thirty three components were identified representing 89.30% of the total oilcomposition. The yield of essential oil was 0.4% and the predominant components were citronellal (14.40%), isogeraniol (6.40%), geraniol acetate (10.20%), nerol acetate (5.10%), caryophyllene (8.10%) and b-caryophyllene oxide (11.00%).

Antibacterial activity of the oil showed the higher activity against all bacterial strains tested. The essential oil extracted from lemon balm can be used to clean theenvironment of reanimation polyvalent and anaesthesia service.

**Keywords:** Melissa officinalis; essential oils; antibacterial activity; nosocomial infections

### Evaluation of the Antioxidant Activity and Nephroprotective Effect of Propolis in Wistar Rats

LaaroussiH.El-HaskouryR.BouddineT.OussaidD.AkdadM.LyoussiB.Laboratory of Physiology, Pharmacology and Environmental Health, Faculty of Sciences, Dhar El Mehraz, University Sidi Mohamed Ben Abdallah, Fez, Morocco

For a long time, the plant kingdom has provided man with the essential resources for his food, his hygiene and health. Propolis is a natural product derived from plant resins and collected by honeybees (workers) to be used as glue and as draught-extruder for bee hives. It is currently used as an ingredient of biopharmaceuticals, candies and as aconstituent of cosmetics, or as a popular alternative medicine for self-treatment of various diseases.

The aim of this work was to determine the antioxydant activity and the nefroprotective effect of ethanolic extract of propolis following treatment with ultravist. To do so, 30 adult male Wistar rats were randomly divided into 6 groups of 5 rats: group I received orally distilled water at a dose of 1 mL/100 g body weight. This group served as a control. Group II received orally 250 mg/Kg /bodyweight of EEP. Group III received intravenous injection of a single dose of D1 ultravist the 8th day (2 mL/Kg/ bodyweight). Group IV received intravenous injection of a single dose D2 ultravist the 8th day (4 mL/Kg/ bodyweight). Group V was pre-treated with EEP (250 mg/kg PO) 7 days prior to exposure to ultravist (2 mg/kg of bodyweight) and 3 days after. Group VI was pre-treated with EEP (250 mg/kg) 7 days prior to exposure to ultravist (4 mg/kg body weight) and 3 days after.

We found that pretreatment with ethanolic extract of propolis leads to a recovery of the basal levels of the key enzymes of the liver function. This result suggests that EEP pretreatment prevents the nephrotoxic effect induced by ultravist. Hence it is proposed to adopt a preventive strategy in the hospital setting.

**Keywords:** resin; propolis; antioxidant activity; nephrotoxicity; nephroprotective effect 

### Hepatoprotective Effect of Ethanolic Extract of Propolis in Wistar Rats

LaaroussiH.El-HaskouryR.OussaidD.BouddineT.AkdadM.LyoussiB.Laboratory of Physiology, Pharmacology and Environmental Health, Faculty of Sciences, Dhar El Mehraz, University Sidi Mohamed Ben Abdallah, Fez, Morocco

Natural substances are becoming increasingly important in therapeutics, propolis constitutes a real chemical plant from which the maximum profit must be taken for the well-being of the population. Has wide range of biological activities which include antimicrobial, antioxidant, anti-inflammatory, anticancer properties and hepatorenale protective effect.

In the present study we were interested in elucidating the hepatoprotective effect of the ethanol extract of propolis following treatment with ultravist contrast agent. 20 rats were used in this experiment, Towards the end of the surgical stage, the rats receive via the jugular vein an infusion with a physiological solution (0.9% NaCl), with a constant flow rate of 25 uL/min/100 g BW. Administrations were performed in all groups as follows : group I received the same perfusion (physiological saline) from start to end of experiment ; group II received during the experimental period (E) the solution of ultravist with a dose of 2 mL/1000 g BW ; group III received during the period (E) extracted from propolis solubilized in physiological water at the dose of 250 mg/10 mL of physiological water ; group IV received EE (250 mg/10 mL) for one hour followed by ULT at the dose of 2 mL/1 Kg PC during the experimental phase (E).

The Intravenous infusion of rats during the experimental phase by ultravist at a dose of 2 mL/kg BW resulted in a net and significant increase in ALAT, ASAT, LDH and PAL at the plasma level (65.55 ± 3.75 (u/L), 70.33 ± 5.10 (u/L), 698.23 ± 11.09 (u/L), 360.67 ± 11.12 (u/L) respectively, whereas the group of rats pretreated with EEP subsequently perfused by ultravist at the Same dose, almost restored the normal plasma levels of these key enzymes of liver function.

In conclusion, the bolus injected EEP has a remarkable hepatoprotective effect, in addition the extract of this product attenuates the toxicity induced by the contrast products, including ultravist.

**Keywords:** propolis; hepatotoxicity; hepatoprotective; contrast products; ultravist

### Assessment of Antimicrobial Activity of Essential Oil of Pulicaria Mauritanica from Morocco against Some Infectious Pathogens 

LboumhamdiAliZniniMohamed*ChibaneE.MajidiLhouLaboratoire des Substances Naturelles & Synthèse et Dynamique Moléculaire, FST, Errachidia, Morocco*Correspondence: m.znini@yahoo.fr

The objective of this study is to evaluate in vitro the antimicrobial activity of *Pulicaria mauritanica* essential oil (EO) against three referenced pathogenic bacteria Escherichia coli (ATCC 25922), *Staphylococcus aureus* (ATCC 25923) and *Pseudomonas aeruginosa* (ATCC 27853) and a yeast *Candida albicans* using the paper disc diffusion and the macro-dillution in liquid medium methods. The results obtained by the first method indicate that this EO showed a strong antimicrobial activity against three strains tested, which *S. aureus* was found to be the most sensitive while *P. aeruginosa* is the most resistant. Furthermore, the results of the second technique reveal that the Minimum Inhibitory Concentrations (MICs) of this EO are on the order of 0.3 and 0.6 mg.mℓ^−1^. Moreover, the results of the report CMB/CMI show that the EO has a bactericidal effect on *S. aureus* and a bacteriostatic and fungistatic effect on *E. coli* and *C. albicans*, respectively.

**Keywords:** Pulicaria mauritanica; Essential oil; carvotanacetone; antimicrobial activity; microbial strains

### Evaluation of Medicago Sativa Growth Inoculated with Rhizobial Strain Rhol1 and/or Arbuscular-Mycorrhizal (AM) Fungi under Stress Conditions

Ben LaouaneR.*1*2*3MeddichA.*1FaghireM.*3OufdouK.*2BechtaouiN.*2El AmeranyF.Ait El MokhtarM.*1WahbiS.*1*1LLaboratoire de Biotechnologie et Physiologie Végétale FSSM-Université Cadi Ayyad. Marrakech*2Laboratoire de Biologie et Biotechnologie des Microorganismes FSSM-Université Cadi Ayyad*3Laboratoire de Biotechnologie Végétale Faculté des Sciences Université Ibn Zohr Agadir

Alfalfa (*Medicago sativa*), is a perennial flowering plant in the family Fabaceae. It is cultivated as an important forage crop in many countries around the world. This plant has been used as a common ingredient in south-west Moroccan (Tinghir) and South Indian cuisine. Alfalfa is rich in many essential vitamins and minerals, including A, D, E, K, and even the full family of B vitamins; biotin, calcium, folic acid, iron, magnesium, potassium …, as well as being very high in protein. This herb is believed to have a direct connection to lowering cholesterol. It is very good at detoxifying and better purifying the blood. It has also beneficial healing properties against bad breath, sore or achy joints, imbalanced skin conditions, and it even increases immune system functionality. This herb acts as an alternative to over the counter pain medicines for headaches or migraines. As a result, consuming alfalfa on a routine basis has an abundance of positive health results. Medicago sativa constitutes the first forage crop in Mediterranean area In Morocco, this crop occupies over 22% of the total area devoted to forage crops. It strongly contributes to socio-economic development of local families. However, water and soil salinity recorded in many world regions is a major environmental factor limiting plant growth and productivity and constitutes an important constraint to alfalfa. In this context, the present study aimed to evaluate the salinity tolerance of Midecago sativa inoculated with Rhizobial strain RhOL1 and/or arbuscular-mycorrhizal (AM) fungi (autochtonous mycorhizal).

**Keywords:** *Medicago sativa*; stress conditions; arbuscular-mycorrhizal; *Rhizobial* strain RhOL1; Growth

### The Cytotoxic Activities of Salvia Officinalis l. and Osmarinus Officinalis l. Leaves Extracts on Human Glioblastoma and Human Neuroblastoma Cell Lines May Be Linked to Their Antioxidant Effects

CHOUKAIRIZineb^1^*LAMRILaila^1^FERRANDEZJosé Manuel^2^FECHTALITaoufiq^1^1Laboratory of Biosciences, Functional, integrated and molecular exploration. Scool of Sciences and Technology-Mohammedia, Hassan II University of Casablanca2Laboratory of “Inteligencia Ambiental”, Polytechnic University of Cartagena, Spain*Correspondence: choukairizineb@gmail.com

*Salvia officinalis L.*(sage) and *Rosmarinus officinalis L.*(rosemary) are two plants that grow spontaneously in some area of morocco, they are used in folk medicine for the treatment of different disorders specially ulcer, diarrhea, rheumatism and inflammation. The objective of this study was to investigate the in vitro anti-tumoral and cytotoxic activities of the methanolic extract prepared from S. Officinalis and R. Officinalis leaves on human glioblastoma and human neuroblastoma cell lines. as well as their antioxidant activity.

The accelerated solvent extract technique was used to obtain the total extract of the plants; then the microculture tetrazolium (MTT) cytotoxic assay was used to evaluate their anti-tumoral activities. the antioxidant potency was evaluated employing various established in vitro systems such as the 1,1-diphenyl-2-picrylhydrazyl (DPPH) free radical scavenging and the ferric reducing antioxidant power (FRAP) assay. This activity was correlated with the phenolic content and the concentration of flavonoids in the plants using spectrophotometric methods.

Ours preliminary results show that methanolic extract of Salvia and rosemary inhibit the glioblastoma cell proliferation; however the plants has no effect on neuroblastoma cell line. In the other hand, the DPPH scavenging assay and the FRAP assay results showed a dose-dependent antioxidant activity of the plants extracts, with an elevated contents of phenols and Flavonoids. The *Salvia officinalis L. and Rosemarinus Officinalis L*. total extract was found to have an anticancer activity which may be linked, probably, to an antioxidant process. In conclusion, our study show interesting results of a potential role of these two plants on the cancer palliation that must be more explored. 

**Keywords:** *Salvia officinalis* L; *Rosmarinus officinalis*; cancer; anti-oxydant; therapeutic effect

### Ethnobotanical Study of Two Medicinal Plants: Vitex Agnus Castus and Anabasis Aretioïdes

AssiaBERRANI^1^*Aicha LRHORFIL.^1^LARBIOuahidi My^2^ZOUARHIMeryem^3^BOUFELLOUSMoncef^1^LellaOuld Abdellahi^1^BENGUEDDOURRachid^1^1Laboratory laboratory biochemistry, biotechnology, health and the environment, Department of Biology, Faculty of Sciences, Ibn Tofail University, Kenitra, Morocco2Laboatory of genetics neuroendocrinology and biotechnology, Department of Biology, Faculty of Sciences, Ibn Tofail University, Kenitra, Morocco3Laboratory of Electrochemistry and Materials Environment (LMEE), Department of Chemistry, Faculty of Sciences, Ibn Tofail University, Kenitra, Morocco*Correspondence: assia.berrani@hotmail.fr

In the aim of the valorization of medicinal and aromatic plants of the Moroccan and Saharein flora, we interested in this ethnobotanical study to two species frequently used by the population local for their virtues: *Vitex agnus castus* of the verbenaceae family, commonly called kharwaâ and the other spéce was *Anabasis aretioids* of the chenopodeaceae family, knowed as Shejra li maidihach errih name.

For this reason, a series of ethnobotanical surveys was carried out in 2015, using a questionnaire, in three regions (Meknes Tafilalet, Gharb Chrarda Bni Hssen, and Rabat Sale Zemmour Zaer). Traditional herbalists and druggists in order to give the information about the medicinal virtues of these plants.

The results obtained shown that the leaves are the most used part of the both plants, moreover the majority of the remedies are prepared in the form of decoction for *Anabasis* and in powder form for *Vitex*.

The diseases treated by these plants were diseases of the digestive tract occupied the first place for *Anabasis* with a rate of 27.67%, followed by use as poison antidotes with a rate of 20.67%. Fur ther more, for *Vitex* the genitourinary affections occupied the first place with a rate of 46.67% followed by dermatological affections with a rate of 20%.

**Keywords:** vitex agnus castus; Anabasis aretioïdes; ethnobotanical; survey; traditional medicine

### Phytochemical Screening and Antioxidant Activity of Celery Seed Essential Oils

Y. El Atki I. Aouam A. Taroq F. El Kamari B. Lyoussi and A.AbdellaouiLaboratory of Physiology Pharmacology and Environmental Health, Department of Biology, Faculty of Sciences Dhar Mehraz ,University Sidi Mohamed Ben Abdellah, B.P. 1796, Atlas, Fez, Morocco

This study was conceived to determine the phytochemical characterization, and antioxidant activity of celery *(Apium graveolens)* seed essential oils. 

The essential oils (EO) of celery seed were isolated by hydrodistillation technique, using a Clevenger-type apparatus. The phytochemical characterization was investigated using gas chromatography-mass spectrometer analysis. The antioxidant activity was evaluated in vitro by two assays namely Free radical scavenging activity against 1,1-diphenyl-2-picrylhydrazyl (DPPH) and Fe2^+^ chelating activity. 

The obtained results showed that the main components detected in celery seed oils were Citronellol (β-sélinène) (35.93%), Epiglobulol (31.36%) and Limonene (7.55%). The two antioxidant assays showed that these oils have a very important antioxidant power confirmed by its richness in polyphenols and flavonoids. Our study suggests that celery seed essential oils have the potential to be used as a natural food preservative.

**Keywords:** essential oils; celery; antioxidant activity; polyphenols; flavonoids

### Overview on the Consumers of Dietetic and Cosmetic Argan Oil

BlazardArmelMarketing and research, University of Lyon, France

The consumption of argan oil beyond the Moroccan grounds is notable in dietetics and cosmetic. The question remains to know if the users have a real knowledge of the benefits of this oil on the health and how they reach related information. Curious to know which would be the social and economic challenges of argan oil benefits on health according to the French populations, we questioned 403 people of «Drôme-Ardèche» in Rhône-Alpes area in France; this includes 142 faithful customers of “stores organic” and “rays organic” in (8) supermarkets, 6 dieticians, 2 nutritionists, 3 estheticians, 100 customers of (15) dietetics offices and 150 customers of (6) wellness centers. This reveals that 65% of the consumers are women (any sector confused). 80% of surveyed consume argan oil in alternation with olive oil according to their dietician recommendations. Among 80%, the rate of the diabetics ones is 40% with 5% of children and 5% of teenagers, 14% of women and 16% of men; 25%, fight obesity and 15% have or tend towards an overload ponderal. The 40% others are in diet because suffer from cancer, 20% suffer celiac diseases, 10% suffer from the gastric diseases and 10% have a structured lifestyle and intended to say “good” of argan oil. The dieticians and nutritionists note the contribution of this oil on health: “anti-oxidant”, “cholesterol fights”, etc except that they do not express themselves on the uses of this oil in curative or preventive therapies. In cosmetic, the users and estheticians note the concrete results on the skin and the hairs. In centers of wellness, argan oil is used as an oil of massage. In conclusion, argan oil is often considered as miracle oil able to do everything. A collaboration between scientists, communicating researchers and professionals would help to collect information on the benefits of this oil. 

**Keywords:** argan oil; economic challenges; social challenges; dietetic; cosmetic; wellness; health; marketing investigations

## The following abstracts were submitted in French and only displayed by Title, Authors and Affiliations.

### Antioxidant Activities and Variations in the Content of Polyphenol and Total Flavonoids of the Essential Oil of Laurus Nobilisl

TaroqAmal*El kamariFatimaEl AtkiYassineAoumImaneLyoussiBadiaaAbdellaouiAbdelfattahLaboratory of Physiology Pharmacology and Environmental Health, Department of Biology, Faculty of Sciences Dhar Mehraz, University Sidi Mohamed Ben Abdellah, B.P. 1796, Atlas, Fez, Morocco*Correspondence: taroq.amal@gmail.com

### Phytochemical Screening and Antibacterial Activity of Rosmarinus Officinalis Essential Oil

TaroqAmal*El kamariFatimaAoumImaneEl AtkiYassineLyoussiBadiaaAbdellaouiAbdelfattahLaboratory of Physiology Pharmacology and Environmental Health, Department of Biology, Faculty of Sciences Dhar Mehraz, University Sidi Mohamed Ben Abdellah, B.P. 1796, Atlas, Fez, Morocco*Correspondence: taroq.amal@gmail.com

### Content of Polyphenols and Flavonoids and Antioxidant Activity of Odorous and Non-Odorous Aqueous Extracts of An Asteraceae: Asteriscus Graveolens (FORSSK) Less

ChibaneEl Mustapha*BoumezzourhAmalLboumhamdiAliZniniMohamedMajidiLhouLaboratoire des Substances Naturelles & Synthèse et Dynamique Moléculaire, Faculté des Sciences et Techniques, Errachidia, Maroc*Correspondence: elmustaphachibane@gmail.com

### Evaluation of the In Vitro Antifungal Activity of Essential Oil and Extracts of Cistus ladaniferus L. Var. Maculatus Dun

BoukiliM.^1^*ChakirS.^1^ChraibiM.^2^Fikri BenbrahimK.^2^HalouiZ.^1^LouziL.^3^EchchgaddaG.^4^1Laboratoire de l’environnement et santé, Université Moulay Ismail Faculté des Sciences, B.P. 11201 Zitoune Meknès, Maroc2Laboratoire de biotechnologie microbienne, Faculté des Sciences et Technique, Université Sidi Mohamed Ben Abdallah, B.P 2202 Fès, Maroc3Centre de laboratoire et pharmacie, Laboratoire de Microbiologie, Hôpital Militaire Moulay Ismail, Meknès, Maroc4Département de Protection des Plantes et de l’Environnement, Ecole Nationale d’Agriculture, Meknès, 50001, Maroc*Correspondence: m.boukili85@gmail.com

### Synthetic Approach of the Bicycle ab Present in Taxol

LachgarM.^1^MraniD.^2^BouachrineM.^3^TabyaouiM.^4^1FST, Errachidia, Maroc2ESTM, Université Moulay Ismaïl, Meknès, Maroc3LMNE, Faculté des Sciences de Rabat, Maroc4Mohammed V University of Rabat , Rabat, Maroc

### Composition in Saturated and Transfatty Acids in Fast-Food Restaurants in Casablanca

El-KardiYounesJafriAliAnideAmalDerouicheAbdelfettahUniversité Hassan II de Casablanca. Faculté des Sciences Ben M'sik. Laboratoire de Biologie et Santé (URAC 34). Unité de Recherche Nutrition Humaine, Casablanca, Maroc

### Contribution to the Study of the Conservative Effect of Certain Aromatic Plants Used as an Additive in the Paste of Dates

AzzaouiB.SakiliD.Université moulay ismail, Faculté des Sciences et Technique Errachidia

### Contribution to the Study of the Antioxidant and Antibacterial Activity of Traditional Midelt’s Vinegar

OusaaidDriss^1^MansouriIsmail^2^RochdiMouad^2^LaaroussiHassan^1^LyoussiBadiaa^1^EnnajiHayat^3^El ArabiIlham^1^1Laboratoire de Pharmacologie Physiologie et Santé Environnementale. Faculté des sciences Dhar El Mahraz, Université Sidi Mohamed Ben Abdellah, Fès, Maroc2Laboratoire d’écologie fonctionnelle et Environnement. Faculté des Sciences et Techniques, Université Sidi Mohamed Ben Abdellah, Fès, Maroc3Laboratoire de sécurité alimentaire et environnemental, Institut pasteur Casablanca, Maroc

### Efficacy of Essential Oils of Origanum Compactum to Control Callosobruchus Maculatus (Coleoptera, Bruchinae)

DouiriLalla Fatima^1^*BouharebHayat^1^lafkihNada^1^GhouatiYasmine^2^MeryemChakir^3^MoumniMohieddine^1^1(Départment de Biologie Faculté des Sciences, /Université Moulay Ismail, PB 11201, Meknès, Maroc)2(Unité de Technologie Alimentaire et Biochimie/National school of Agriculture, B P S/40 50000 Meknès Maroc)3(Départment de Biologie Faculté des Sciences, /Université Ibn Tofail, BP: 14000, Kenitra, Maroc)*Correspondence: fatimadouiri@yahoo.fr

### Study of the Insecticidal Activity of Taxus Baccata of the Val D'ifrane on Sitophilus Oryzae (Coleoptera: Curculionidae)

ElhourriMohammed^1^El idrissiMostafa^1^AmechrouqAli^1^LemrhariAdrae^2^GhouatiYasmine^3^1Laboratoire de Chimie Moléculaire et Substances Naturelles, Université Moulay Ismail, Faculté des Sciences, B.P. 11201, (Zitoune), Meknès, Maroc2Laboratoire de Biotechnologie des Plantes et de Biologie Moléculaire, Université Moulay Ismail, Faculté des Sciences, B.P. 11201, (Zitoune), Meknès, Maroc3Ecole Nationale d’Agriculture, Unité de Technologie Alimentaire et de Biochimie BP S/40 Meknès. Maroc. med.elhourri@gmail.com

### Chemical Composition and Antibacterial Activity of Essential Oils of Rosmarinus Officinalis

AouamImane*El AtkiYassineTaroqAmalEl KamariFatimaBadiaaLyoussiAbdellaouiAbdelfattahLaboratoire de Physiologie, Pharmacologie et Santé Environnementale, Faculté des Sciences Dhar El-Mehraz, Université Sidi Mohamed Ben Abdallah, Fès, Maroc*Correspondence: imaneaouam@hotmail.com

### Use of Essential oils in the Treatment of Post-Traumatic Tendon-Muscular and Ligamentous Lesions in Athletes

ZakariyaI.MoutaouakkilY.IfezouaneJ.BennaniI.NejjariR.Laboratoire de pharmacognosie; faculté de médecine et de pharmacie de Rabat

### Evaluation of the Therapeutic and Toxicological Knowledge of 20 Herbalists in the Rabat area of Morocco on the Top 5 Plants Most Reported to the National Center for Pharmacovigilance in Morocco

ZakariyaI.IfezouaneJ.AddaouiA.MoutaouakkilY.NejjariR.BouslimaneY.

### Antioxidant and Antiinflammatory Activities of Thymelaea Lythroides

BerkiksInssaf^1^*SghirZ.MesfiouiA.MarmouzziI.OuichouA.AkhwayriO.lhessnaouiA.BouslhamR.NakkachR.BahbitiY.El hessniA.^1^1Laboratory of Genetic, Neuroendocrinology and Biotechnology—Faculty of Sciences, University Ibn Tofail, Kenitra, Morocco*Correspondence: berkiksinssaf@gmail.com

### Protective Effect of Rosmarinus officinalis and Eucalyptus globulus on the Protozoan Tetrahymena thermophila

BouchraEl khalfi^1^DaoudaMar Papa^1^ChemsiIsa^1^*AbdelazizSoukri^1^1Laboratoire de Physiopathologie Génétique Moléculaire & Biotechnologie, Faculté des Sciences Ain Chock de Casablanca, Maroc*Correspondence: isa.el.chemsi@gmail.com

### Study of the Effect of Organic Walnut Fractions (Juglans Regia) on Platelet Aggregation and In Vitro Plasma Coagulation in Rats

AMIROUAsmaeAZIZMohammedBNOUHAMMohamedZIYYATAbderrahimMEKHFIAbdelkhaleq LEGSSYER et Hassane*Université Mohammed Premier, Faculté des Sciences, Laboratoire de Physiologie, Génétique et Ethnopharmacologie, Oujda, Maroc*Correspondence: hmekhfi@yahoo.fr

### Synthesis of New r - (+) - Pulegone Derivatives and Study of Their Impact on Fungi Penicillium Expansum, Rhizopus Stolonifer and Alternaria sp

AhmadOUBAIRRachidFIHIDrissCHEBABELaboratoire des Substances naturelles & Synthèse et Dynamique Moléculaire

### Study of the Insecticidal Activity of Taxus Baccata of the Val D'ifrane on Sitophilus Oryzae (Coleoptera: curculionidae)

ElhourriMohammed^1^*El idrissiMostafa^1^AmechrouqAli^1^LemrhariAdrae^2^GhouatiYasmine^3^1Laboratoire de Chimie Moléculaire et Substances Naturelles, Université Moulay Ismail, Faculté des Sciences, B.P. 11201, (Zitoune), Meknès, Maroc2Laboratoire de Biotechnologie des Plantes et de Biologie Moléculaire, Université Moulay Ismail, Faculté des Sciences, B.P. 11201, (Zitoune), Meknès, Maroc3Ecole Nationale d’Agriculture, Unité de Technologie Alimentaire et de Biochimie BP S/40 Meknès. Maroc*Correspondence: med.elhourri@gmail.com

### Evaluation of the Consumption of Olive Oil in an Infantile Population, Province of Errachidia

AzekourK.BidiA.OutalebZ.MachraouiS.NasriI.EddouksM.El BouhaliB.Département de biologie, Faculté des Sciences et Techniques, Université Moulay Ismail, Errachidia, MarocDépartement de biologie, Faculté des Sciences et Techniques, Université Hassan II, Mohammedia, Maroc

### Study of the Healing Properties of the Micocoulier Seeds (Celtis Australis)

Filali-AnsariNajoieEcharrafiSoukainaEl MalikiSoukainaEl KhyariSaidEl AbbouyiAhmedLaboratoire de Biochimie, Nutrition et Valorisation des Ressources Naturelles (LBNVRN), Faculté des Sciences, BP20, El Jadida, Maroc

### Place of Medicinal Plants in the Treatment of Diabetes in the Middle Sebou Region (Atlantic morocco)

SalhiS.^1^*Ben AkkaF.^1^Filali-ZegzoutiY.^2^DouiraA.^1^et ZidaneL.^1^1Laboratoire de Biodiversité et Ressources Naturelles, Faculté des Sciences de Kénitra2Équipe de recherche : Biologie, Environnement & Santé, FST-Errachidia*Correspondence: salhisouad@gmail.com

### Evaluation of the Chemical Composition and the Antibacterial Potential of Some Essential Oils in Biological Control against Pathogens Agents Responsible for Human infections

SabiAsmaa*El KhalfiBouchraErrachidiFaouziSoukriAbdelazizLaboratoire de Physiopathologie Génétique Moléculaire et Biotechnologie, Faculté des Sciences Ain Chock, Km 8 route d’El Jadida, B.P. 5366 Mâarif. Casablanca, Morocco*Correspondence: sabiasmaa@gmail.com

### Antioxidant and Antiinflammatory Activities of Thymelaea Lythroides

BerkiksInssaf*SghirZ.MesfiouiA.MarmouzziI.OuichouA.AkhwayriO.ElhessnaouiA.BouslhamR.NakkachR.BahbitiY.El hessniA.Laboratory of Genetic, Neuroendocrinology and Biotechnology—Faculty of Sciences, University Ibn Tofail, Kenitra, Morocco*Correspondence: berkiksinssaf@gmail.com

### Chemical Composition and Antibacterial Activity of Essential Oils of Rosmarinus Officinalis

AOUAMImane*ATKIYassine ELTAROQAmalKAMARIFatima ELLYOUSSIBadiaaABDELLAOUIAbdelfattahLaboratoire de Physiologie, Pharmacologie et Santé Environnementale, Faculté des Sciences Dhar El-Mehraz, Université Sidi Mohamed Ben Abdallah, Fès, Maroc*Correspondence: imaneaouam@hotmail.com

### Antioxidant Effect of Essential Oils of Aromatic and Medicinal Plants in Southern Morocco

TaoufikF.^1^*ZineS.^1^HamdouchA.^2^EL HadekM.^1^AsdadiA.^2^Idrissi HassaniL.M.1Laboratoire de Génie des Procédés, Faculté des Sciences d’Agadir, Université Ibn Zohr, B.P 28/S, Agadir, Maroc2Laboratoire de Biotechnologies Végétales, Equipe Planta Sud, Faculté des Sciences d’Agadir, Université Ibn Zohr, B.P 28/S, Agadir, Maroc*Correspondence:taoufik.fatima@gmail.com

### Phytochemical Study and Evaluation of the Antihyperglycemic and Antihyperlipidemic Activity of the PISTACIA Atlantica Oil of the Region of Errachidia

HajjiL.^1^KhalloukiF.^2^ZemzoumiM.^1^EL OuadiF.^1^ChaâchouâyH.Filali-ZegzoutiY.^3^HALOUIZ.^4^EddouksM.^1^et El GhissassiM.^5^1Equipe de recherche Physiologie et Pharmacologie Endocrinienne FST Errachidia2Equipe de recherche Substances Naturelles & Agro-ressources FST Errachidia3Equipe de recherche Biologie, Environnement & Santé FST Errachidia4Lab. Environnement & Santé, FS, Kénitra5Equipe de recherche Neurosciences FS Kénitra

### Contribution to the Study of the Antioxidant Activity of Beeswax

BouddineT.LaaroussiH.OussaidD.AkdadM.LyoussiB.Laboratory of Physiology, Pharmacology and Environmental Health, Faculty of Sciences, Dhar El Mehraz, University Sidi Mohamed Ben Abdallah, Fez, Morocco

### Consumption of Dates Versus Obesity: A Cross-Sectional Study among Women from the SOUTH east of Morocco

BidiAminaOutalebZahraAzekourKarimaEddouksMohamedEl BouhaliBachirDépartement de Biologie, Faculté des Sciences et Techniques Errachidia, Université Moulay Ismail, Maroc

### Study of the Effect of Lavandula Stoechas on Neuropathic Pain in Laboratory Animals

FerehanHind^1^AboufatimaRachida^2^ChaitAbderrahman^1^,^1^1Laboratoire de Neurobiologie Pharmacologie et Comportement. Faculté des Sciences Semlalia. Université Cadi Ayyad2Laboratory of Biological Engineering. Natural Substances, Cellular and Molecular Immuno-pharmacology Group.Sultan Moulay Slimane University; Faculty of Science and Technology, Béni-Mellal

## 5. Conclusions

This first meeting on argan oil brings several information on this oil which was often compared with olive oil. The composition of argan oil was presented as well as its biological properties on different cells and animal models. Clinical studies were also presented underlying the benefits of this oil mainly on the lipid and inflammatory status. Promising results on the benefits of argan oil on nerve cells and neurological diseases were also shown. Altogether, the data exposed supported the benefits of argan oil on human health. However, additional works are still required to clarify its properties and its benefits. The authors attending to the meeting or not have the opportunity to publish their work on argan oil or on other oils under the form of research papers or reviews in a special issue of IJMS entitled ‘The Beneficial Effects of Plant Oil on Human Health’.

## 6. Awards Winners 

**Dr. A. Zarrouk** (Oral communication)

*Comparison* *of the Contents of the Main Biochemical Compounds and the Antioxidant Activity of Argan Oil, Olive Oil, Silybum Marianum Seed Oil, Nigella Seed Oil and Colza Oil*

Laboratoire ‘Nutrition, Aliments Fonctionnels et Santé Vasculaire’, UR12ES05 Université de Monastir, Monastir, Tunisia 

Equipe ‘Biochimie du Peroxysome, Inflammation et Métabolisme Lipidique’ EA 7270/Université de Bourgogne Franche Comté/Inserm, Dijon, France

Laboratoire de Biochimie, Faculté de Médecine, Sousse, Tunisia.

**Mlle. M. Bakour** (Poster presentation)

*Argan* *oil and Clove Essential Oil Improves Biochemical and Histological Change by Reducing Oxidative Stress Induced by Hydrogen Peroxide in Wistar Rats*

Laboratory of Physiology-Pharmacology-Environmental Health, Faculty of Sciences Dhar El Mehraz, University Sidi Mohamed Ben Abdallah, Fez, Morocco

## 7. Conferences Photos of the Meeting

**Figure 1:**
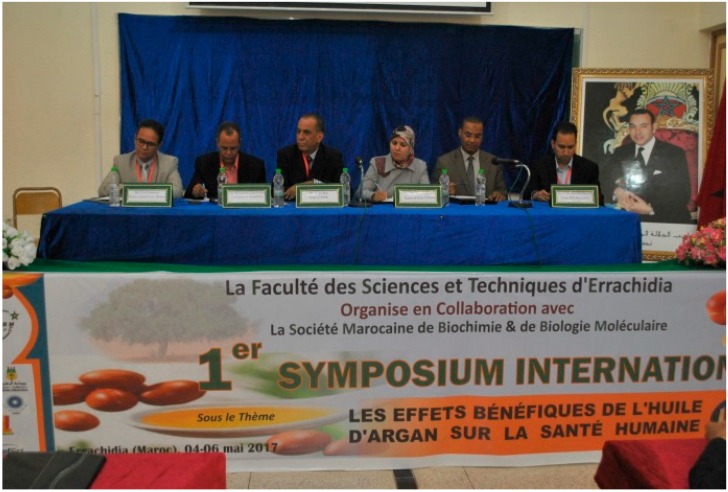
Opening session of the 1st symposium on argan oil by the officials.

**Figure 2:**
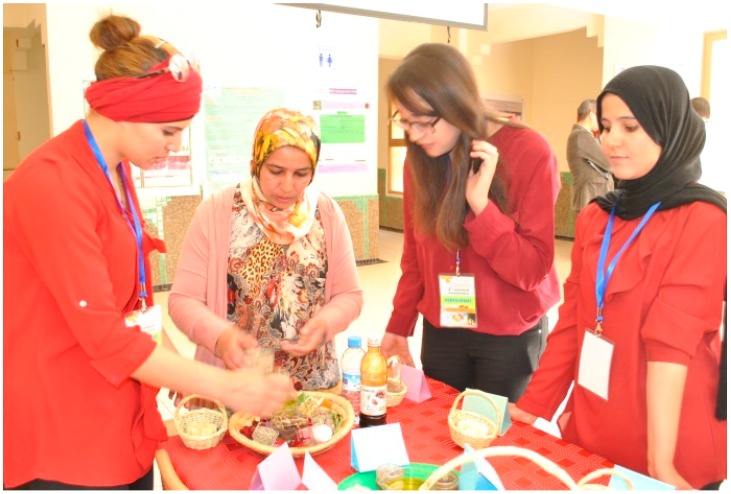
Presentation of food products and cosmetics with bases of Argan oil.

**Figure 3:**
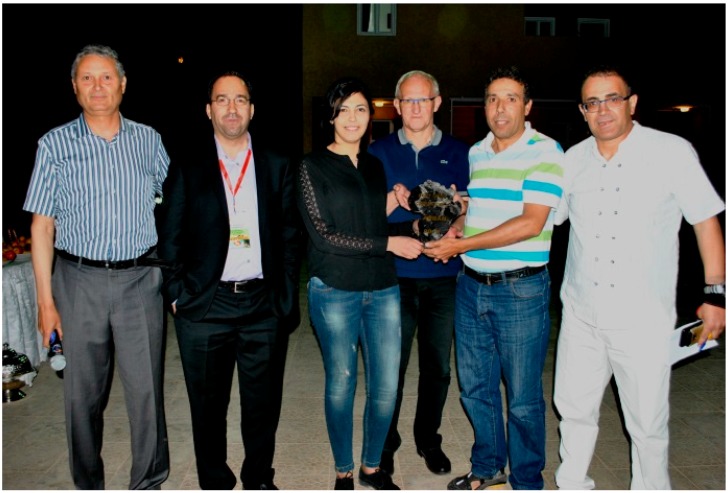
Award of the best oral presentation (Dr A. Zarrouk (3rd on the left), Ass. Prof, Univ. Monastir (LR NAFS-12ES05)/Univ. Sousse (Lab. Biochemistry), Tunisia and Univ. Bourgogne Franche-Comt? (Lab. Bio-PeroxIL/EA7270/Inserm, France) during the gala evening.

**Figure 4:**
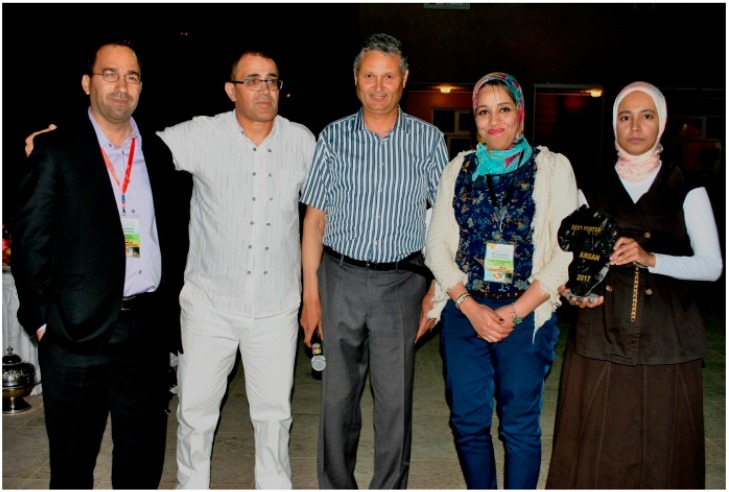
Award of the best poster (Ms M. Bakour (1st on the right), Ph.D student, Laboratory of Physiology-Pharmacology-Environmental Health, Faculty of Sciences Dhar El Mehraz, University Sidi Mohamed Ben Abdallah, Fez, Morocco) during the gala evening. The award has been delivered by the members of the organizing committee: Prof Dr. A. El Midaoui, Pr. Y. Filali-Zegzouti and Pr. L. El Rhaffari.

**Figure 5:**
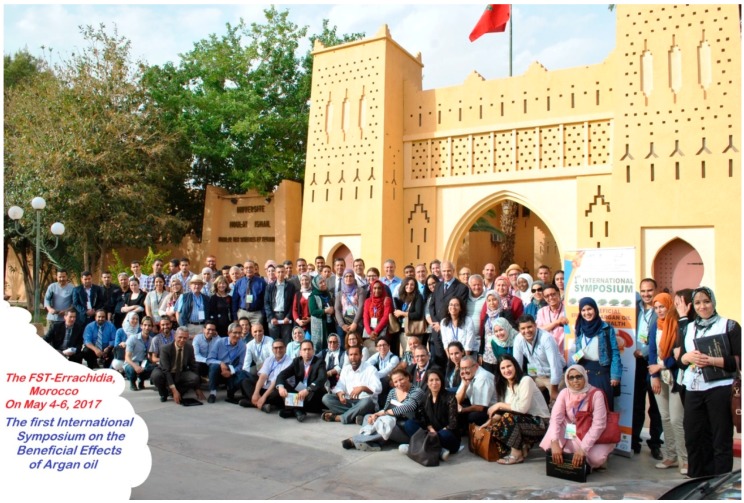
Symposium participants.

